# Identification of a novel cAMP dependent protein kinase A phosphorylation site on the human cardiac calcium channel

**DOI:** 10.1038/s41598-017-15087-0

**Published:** 2017-11-09

**Authors:** Henrietta Cserne Szappanos, Padmapriya Muralidharan, Evan Ingley, Jakob Petereit, A. Harvey Millar, Livia C. Hool

**Affiliations:** 1 School of Human Sciences, University of Western Australia, Crawley, Western Australia Australia; 2 Harry Perkins Institute of Medical Research and Centre for Medical Research, University of Western Australia, Nedlands, Western Australia Australia; 3 School of Veterinary and Life Sciences, Murdoch University, Murdoch, Western Australia Australia; 4 ARC Centre of Excellence in Plant Energy Biology, University of Western Australia, Crawley, Western Australia Australia; 5 Victor Chang Cardiac Research Institute, Darlinghurst, New South Wales Australia

## Abstract

The “Fight or Flight” response is elicited by extrinsic stress and is necessary in many species for survival. The response involves activation of the β-adrenergic signalling pathway. Surprisingly the mechanisms have remained unresolved. Calcium influx through the cardiac L-type Ca^2+^ channel (Ca_v_1.2) is absolutely required. Here we identify the functionally relevant site for PKA phosphorylation on the human cardiac L-type Ca^2+^ channel pore forming α1 subunit using a novel approach. We used a cell free system where we could assess direct effects of PKA on human purified channel protein function reconstituted in proteoliposomes. In addition to assessing open probability of channel protein we used semi-quantitative fluorescent phosphoprotein detection and MS/MS mass spectrometry analysis to demonstrate the PKA specificity of the site. Robust increases in frequency of channel openings were recorded after phosphorylation of the long and short N terminal isoforms and the channel protein with C terminus truncated at aa1504. A protein kinase A anchoring protein (AKAP) was not required. We find the novel PKA phosphorylation site at Ser1458 is in close proximity to the Repeat IV S6 region and induces a conformational change in the channel protein that is necessary and sufficient for increased calcium influx through the channel.

## Introduction

It is well recognized that many species are able to evade a threat by way of activation of the “Fight or Flight” response. The sympathetic nervous system drives this through activation of the β-adrenergic receptor signaling pathway that results in positive chronotropic, inotropic and lusitropic effects in the heart. At the level of cardiomyocytes, β-adrenergic receptor (β-AR) stimulation leads to an increase in cyclic AMP (cAMP) production by adenylate cyclase coupled to G_S_ proteins. The locally increased cAMP level activates cAMP- dependent protein kinase A (PKA) that phosphorylates many target proteins^1–3^ ultimately increasing the intracellular calcium level and contractile force.

The Ca_v_1.2 protein (also known as the α1 C subunit) is the pore forming and ion conducting subunit of the cardiac L-type Ca^2+^ channel and the main route for calcium influx into cardiac myocytes. Calcium influx through the channel initiates contraction and regulates excitation. Although β-AR-mediated stimulation of Ca_v_1.2 has been studied for decades, and PKA is a recognized downstream effector of β-AR signaling, the molecular mechanism responsible for PKA regulation of the channel remains unresolved.

Many studies have suggested that the C terminus of the Ca_v_1.2 is the target for PKA-dependent phosphorylation. The distal carboxyl terminus (DCT) has been demonstrated to impose an autoinhibitory effect on channel function^4^, contains the sites for calcium and calmodulin binding, and interaction with A kinase anchoring proteins (AKAPs)^5^. AKAP is responsible for proper compartmentalization of PKA in cardiac myocytes^6–9^, and binds to Ca_v_1.2 channels via the leucine zipper motif in the DCT^7,10^ but *in vivo* studies have failed to prove a direct effect of AKAPs on cardiac contractility^11,12^. It has been suggested that the physiologically cleaved C terminal α1 C fragment remains functionally associated with the channel and is responsible for autoregulation^4,13,14^. Deletion of the Ca_v_1.2 α1 DCT at G1796 reportedly caused a loss of β-adrenergic regulation when adenylyl cyclase was directly activated using forskolin in mouse embryonic ventricular myocytes^15^. Truncation of DCT also caused prenatal or neonatal death, due to incorrect membrane insertion of the channel, leading to proteasomal degradation of the truncated protein^15,16^.

Serine 1928 in the DCT was first identified as the main target for PKA-dependent phosphorylation in rabbit heart^17^. Since then, it has been concluded that serine 1928 is not essential for PKA-mediated stimulation of Ca_v_1.2 activity^18,19^. Serine 1700 and threonine 1704 in the proximal C terminal domain of cardiac Ca_v_1.2 channels were also identified as phosphorylation sites^20,21^. Both serine 1700 and threonine 1704 are conserved in the skeletal muscle Ca_v_1.1 channels and serine 1575 and threonine 1579 have been identified as substrates for cAMP-dependent protein kinase. Serine 1575 can be modified by calcium/calmodulin-dependent protein kinase II (CaMKII) in neurons^22^. Serine 1575 and threonine 1579 in Ca_v_1.1 channels and their homologs in Ca_v_1.2 channels are proposed to be located at a regulatory interface between the distal and proximal C terminal domains^22^. More recently it has been demonstrated that serine 1700 is not necessary for β-adrenergic responses in a mouse model^23^ but is necessary for physiological regulation of the channel^24^.

Controversies in phosphorylation studies may originate as a result of the complexity of the β-AR signaling pathway, the heterogeneity of calcium channels in the models tested^25^, alternative splicing^26^, and post-translational proteolytic cleavage at alanine 1800 in the DCT that occurs *in vivo*^14^. Choosing the most appropriate model system is pivotal for comparative studies. However limited knowledge of the β-AR signaling pathway makes it difficult for its full reconstitution in heterologous expression systems^27^.

In this study, we used a direct approach to study the human cardiac Ca_v_1.2 channel by reconstituting the channel protein into artificial liposomes. This cell-free system allows for the measurement of changes in ion-conducting channel function as a result of direct modification of the channel protein measured as single channel currents using patch-clamp technique. The major benefit of this functional assessment compared with the use of expression systems is that channel function is studied in isolation from the auxiliary subunits of the channel complex and the regulatory pathways present in the cell^28^.

We find that serine 1458 in the truncated C terminal short-NT isoform of the human cardiac Ca_v_1.2 channel (corresponding to serine 1535 in Q13936-1) is the target for PKA phosphorylation. Homologous serine 1517 in murine neurons^29^ and serine 1570 in rabbit heart^30^ mediate Ca^2+^ current facilitation. CaMKII shares the same consensus binding site with PKA, and can phosphorylate and modify the function of voltage-dependent calcium channels^31^. It has been suggested that simultaneous activation of these two intracellular signaling pathways contributes to the ‘Fight or Flight’ response^32^. Our results demonstrate that phosphorylation of serine 1458 by PKA is necessary and sufficient to increase open probability of Ca_v_1.2. This is likely to be due to the close proximity of the serine to the ion pore forming region of the channel. CaMKII further potentiates the increase in open probability as a result of binding to the DCT in the full length channel protein.

## Results

### PKA phosphorylation of the full length Ca_v_1.2 (long-NT) isoform alters function

**Figure 1:**
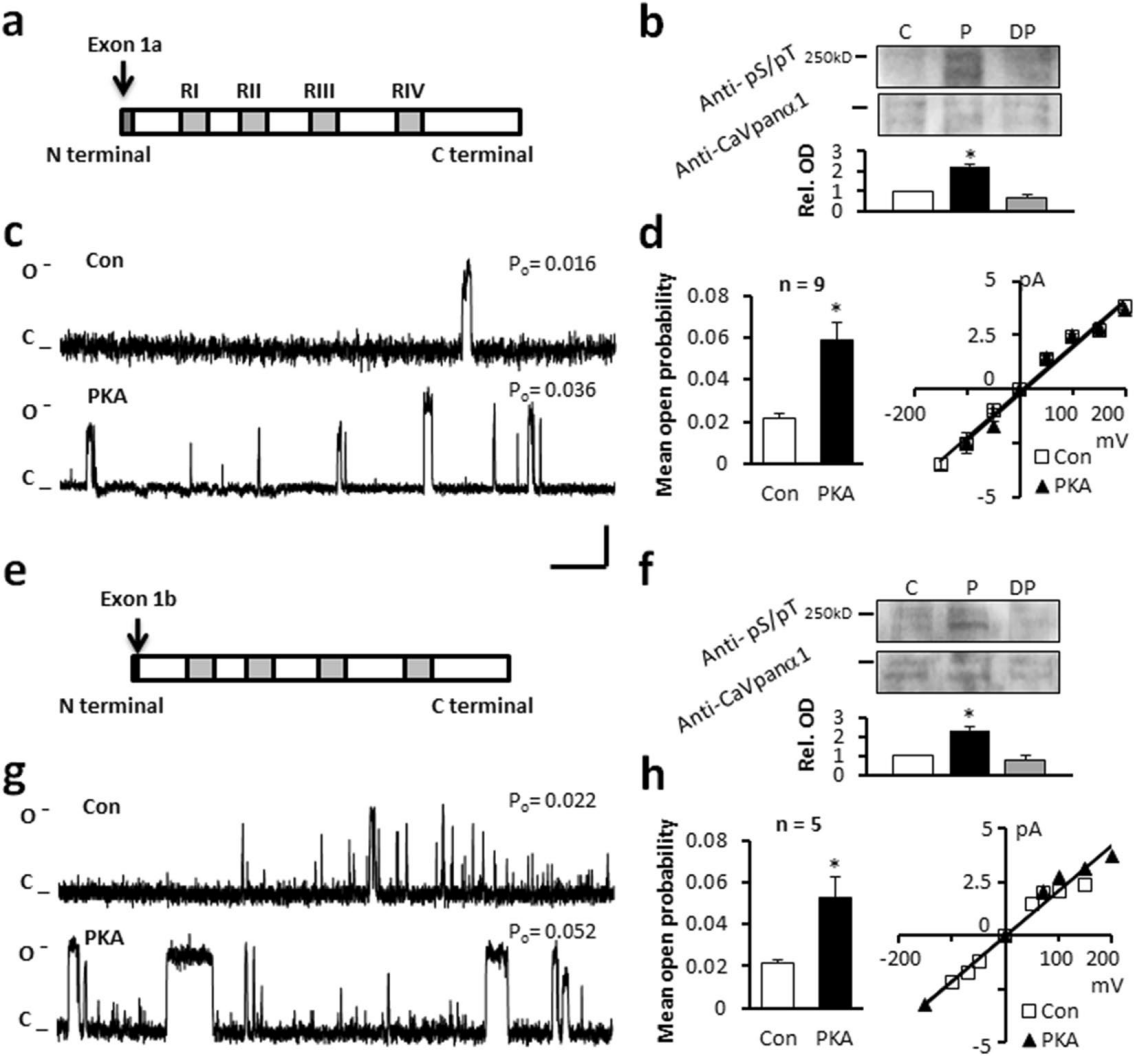
The effect of PKA on the human long-NT and short-NT isoforms of Ca_v_1.2. Schematics of human long-NT (**a**) and short-NT isoforms (**e**) indicating transmembrane repeats (RI-IV, grey bars) distinct exons 1a and 1b, and the amino (N) and carboxyl (C) terminals. (**b,f**) Immunoblots of the Ca_v_1.2 channel protein indicated at ~240 kDa band probed with Anti-CaVpanα1 antibody. Anti-phosphoSer/Thr PKA substrate specific antibody (Anti-pS/pT) demonstrates phosphorylation after *in vitro* PKA phosphorylation (P). Subsequent dephosphorylation by PP2A (DP) removed the signal (C: control). Semi-quantitative western blot analysis demonstrated below (Rel. OD: relative optical density). Full-length blots are presented in Supplementary Figure 2. Representative single channel currents of the long-NT (**c**) and short-NT isoform (**g**) recorded at +150 mV in the absence (Con) and presence of PKA in the same patch (**d,h**) The current-voltage (I–V) relationship (right) and the mean ± SEM channel open probability (P_o_) for currents recorded in the absence and presence of PKA (left). n: number of single channel patches, vertical scale bar indicates 2 pA, horizontal bar indicates 100 ms. *p < 0.05 PKA vs Con.

The Ca_v_1.2 channel is approximately 240 kDa (Fig. 1 and Suppl. Figure 1a) composed of 4 homologous transmembrane domains (Repeat I-IV), a cytoplasmic N and C terminus. When reconstituted in liposomes the Ca_v_1.2 protein forms a functional ion channel (Fig. 1). Substitution of Ba^2+^ for Ca^2+^ in the symmetrical bath and pipette solutions supports current through L-type Ca^2+^ channels, eliminates calcium current facilitation and slows inactivation. The dihydropyridine agonist BayK(-) was used to promote channel openings. We confirmed the properties of the channel by voltage-stepping from −150 mV to +150 mV and measuring the magnitude of the current and its sensitivity to the L-type Ca^2+^ channel antagonist nisoldipine. The magnitude of the outward single channel current at 150 mV was approximately 2.5 pA, decreased as voltage approached 0 mV, reversed at 0 mV (consistent with symmetric solutions) with a slope conductance of approximately 21.3 pS (Fig. 1d) similar to our previously reported work^28^.

During control conditions, the open probability of the channel (P_o_) was 0.022 ± 0.002 (n = 7) and the mean open dwell time was 23.0 ± 3.4 ms (Table 1), consistent with long channel openings associated with binding by BayK(-). In the presence of PKA the mean open probability increased to 0.059±0.008 (Fig. 1d), without significant change in mean open dwell time (22.1 ± 1.8 ms) or single channel current amplitude (2.5 ± 0.2 in control and 2.6 ± 0.1 pA during PKA application, Table 1). PKA did not further increase mean open dwell time presumably because the presence of BayK(-) activated mode 2 openings. To further confirm this, we tested the effect of PKA on single channel currents in the absence of BayK(-). Under these conditions 95.3 ± 2.4% of the time the channel exhibited mode 0 or mode 1 openings. After application of PKA the incidence of mode 2 openings increased from 4.7 ± 2.4% to 30.9 ± 4.9% indicating that the channel was shifting from mode 0 or 1 to mode 2 gating (n = 6). The shift to mode 2 gating can also be seen during acute β-adrenergic activation *ex vivo*, or *in vivo* in the failing heart, when phosphorylation levels of the channel are increased. It has been shown that dihydropyridine agonists have no effect under these conditions. Dihydropyridines bind on Ca_v_1.2 at S5 and S6 transmembrane domain at Repeat III and S6 at Repeat IV and the proximal C terminus^29,33^.

**Table 1:** Single channel properties of Ca_v_1.2 channel forms recorded during different treatments.

	treatment	open probability (Po)	amplitude (pA)	mean dwell time (ms)	N
**long-NT**	—	0.022 ± 0.002	2.5 ± 0.2	23.0 ± 3.4	7
	PKA	0.059 ± 0.008*	2.6 ± 0.1	22.1 ± 1.8	
	St Ht31	0.025 ± 0.004	2.5 ± 0.1	23.7 ± 2.2	8
	St Ht31 + PKA	0.060 ± 0.010*	2.5 ± 0.1	25.8 ± 2.3	
	CaMKII	0.041 ± 0.007*	2.5 ± 0.2	25.3 ± 4.3	5
	CaMKII + PKA	0.073 ± 0.008*^#^	2.5 ± 0.1	23.4 ± 3.3	
**short-NT**	—	0.022 ± 0.002	2.6 ± 0.2	22.2 ± 1.5	5
	PKA	0.053 ± 0.010*	2.5 ± 0.2	22.0 ± 3.5	
**S1928A short-NT**	—	0.025 ± 0.002	2.6 ± 0.1	21.7 ± 2.1	7
	PKA	0.057 ± 0.004*	2.4 ± 0.1	21.9 ± 1.9	
**truncC**	—	0.045 ± 0.007	2.5 ± 0.1	24.6 ± 3.6	15
	PKA	0.156 ± 0.022*	2.6 ± 0.1	25.7 ± 2.9	
	PKI	0.034 ± 0.005	2.6 ± 0.2	23.9 ± 3.4	7
	PKI + PKA	0.034 ± 0.009	2.6 ± 0.2	24.2 ± 3.7	
	—	0.045 ± 0.007	2.6 ± 0.1	22.9 ± 2.2	8
	PP2A	0.037 ± 0.006	2.7 ± 0.1	22.6 ± 3.1	
	PP2A	0.034 ± 0.005	2.6 ± 0.2	23.9 ± 3.4	4
	PP2A + PKA	0.034 ± 0.009	2.6 ± 0.2	24.2 ± 3.7	
	PKA	0.109 ± 0.022	2.4 ± 0.2	22.7 ± 4.1	6
	PKA + PP2A	0.042 ± 0.013	2.4 ± 0.2	25.3 ± 4.3	
	FMP-API-1	0.042 ± 0.012	2.5 ± 0.1	23.4 ± 4.0	5
	FMP-API-1 + PKA	0.145 ± 0.018^‡^	2.6 ± 0.2	24.0 ± 2.5	
	CaMKII	0.049 ± 0.007	2.5 ± 0.2	23.3 ± 1.5	8
***truncC mutants***					
**quadruple mutant**	—	0.046 ± 0.010	2.5 ± 0.2	21.5 ± 3.1	7
	PKA	0.052 ± 0.007	2.5 ± 0.2	21.7 ± 3.2	
**S436A**	—	0.045 ± 0.009	2.4 ± 0.3	23.5 ± 3.8	6
	PKA	0.121 ± 0.016*	2.4 ± 0.1	24.6 ± 2.9	
**S754A**	—	0.043 ± 0.007	2.4 ± 0.2	24 ± 3.2	7
	PKA	0.124 ± 0.029*	2.5 ± 0.2	23.5 ± 3.2	
**S834A**	—	0.040 ± 0.007	2.7 ± 0.2	24.9 ± 3.6	6
	PKA	0.105 ± 0.025*	2.6 ± 0.2	23.4 ± 4.8	
**S1458A**	—	0.043 ± 0.007	2.5 ± 0.1	23.3 ± 4.5	5
	PKA	0.052 ± 0.010	2.4 ± 0.1	23.2 ± 3.2	
	CaMKII	0.041 ± 0.006	2.4 ± 0.1	22.8 ± 5.4	6

N = number of experiments, *p < 0.05 PKA or CaMKII vs Con, ^#^p < 0.05 CaMKII + PKA vs CaMKII alone, ^‡^p < 0.05 FMP-API-1 + PKA vs FMP-API-1 alone.

Semi-quantitative densitometry was performed normalizing the detected phospho-Ser/Thr PKA substrate antibody specific chemiluminescent signal to the signal of channel specific anti-Ca_v_ panα1 antibody. *In vitro* phosphorylation of the protein by PKA resulted in 2.2 ± 0.1 times increase in normalized relative optical density. Subsequent application of PP2A, a major phosphatase acting on L-type Ca^2+^ channels in human heart^34^, sharing substrate specificity with PKA removed the signal (Fig. 1b, Table 2).

**Table 2:** Western blot analysis. Normalized OD values from semi-quantitative immunoblot analysis for all channel protein form.

	treatment	normalized OD values from immunoblots	phosphate groups per molecule predicted from fluorescent phosphoprotein detection
**long-NT**	—	1.0 ± 0.1	1.2 ± 0.1
	PKA	2.1 ± 0.1*	2.6 ± 0.3*
	PKA then PP2A	0.8 ± 0.1	1.3 ± 0.5
**short-NT**	—	1.0 ± 0.1	0.9 ± 0.1
	PKA	2.2 ± 0.2*	2.3 ± 0.3*
	PKA then PP2A	0.8 ± 0.3	0.8 ± 0.3
**S1928A short-NT**	—	1.1 ± 0.1	0.7 ± 0.2^$^
	PKA	2.3 ± 0.1*	1.8 ± 0.2*
	PKA then PP2A	0.8 ± 0.2	1.0 ± 0.3
**truncC**	—	1.1 ± 0.1	1.0 ± 0.1
	PKA	2.7 ± 0.2*	2.2 ± 0.1*
	PKA then PP2A	1.2 ± 0.3	1.2 ± 0.2
***truncC mutants***			
**quadruple mutant**	—	1.1 ± 0.1	1.2 ± 0.1
	PKA	1.2 ± 0.2^#^	1.2 ± 0.1^#^
	PKA then PP2A	0.8 ± 0.1	1.0 ± 0.2
**S436A**	—	1.1 ± 0.1	1.0 ± 0.1
	PKA	2.6 ± 0.3*	2.1 ± 0.2*
	PKA then PP2A	0.8 ± 0.1	0.9 ± 0.1
**S754A**	—	1.0 ± 0.1	1.2 ± 0.1
	PKA	2.5 ± 0.1*	2.5 ± 0.1*
	PKA then PP2A	0.6 ± 0.1*	1.3 ± 0.2
**S834A**	—	1.0 ± 0.1	1.3 ± 0.2
	PKA	2.5 ± 0.1*	2.4 ± 0.3*
	PKA then PP2A	0.8 ± 0.2	1.1 ± 0.1
**S1458A**	—	1.1 ± 0.1	1.1 ± 0.1
	PKA	1.1 ± 0.1^#^	1.2 ± 0.1^#^
	PKA then PP2A	0.8 ± 0.2	1.2 ± 0.1

*p < 0.05 PKA vs Con, ^#^p < 0.05 quadruple and S1458A mutant truncC vs truncC isoform under same condition.

### Short and long-NT isoforms of the Ca_v_1.2 channel respond similarly to protein kinase A

There are two alternative splice variants of the N terminus of the Ca_v_1.2 channel in human heart. Exon 1a codes for the initial 46 amino acids in the N terminus of the long N terminal (long-NT, Fig. 1a) variant and exon 1b codes for 16 amino acids in the short-NT variant (Fig. 1e) of the channel pore forming subunit. We tested whether the short-NT isoform, also lacking the exon 45 encoding region responded in a similar manner to PKA phosphorylation as measured in the long-NT isoform (Fig. 1f–h, Table 1 and Table 2). Our data indicate that the short and long-NT isoforms respond similarly to PKA and exon 1a and exon 45 do not encode the critical serines.

### The C terminal domain and specifically serine 1928 are not necessary for PKA phosphorylation

**Figure 2:**
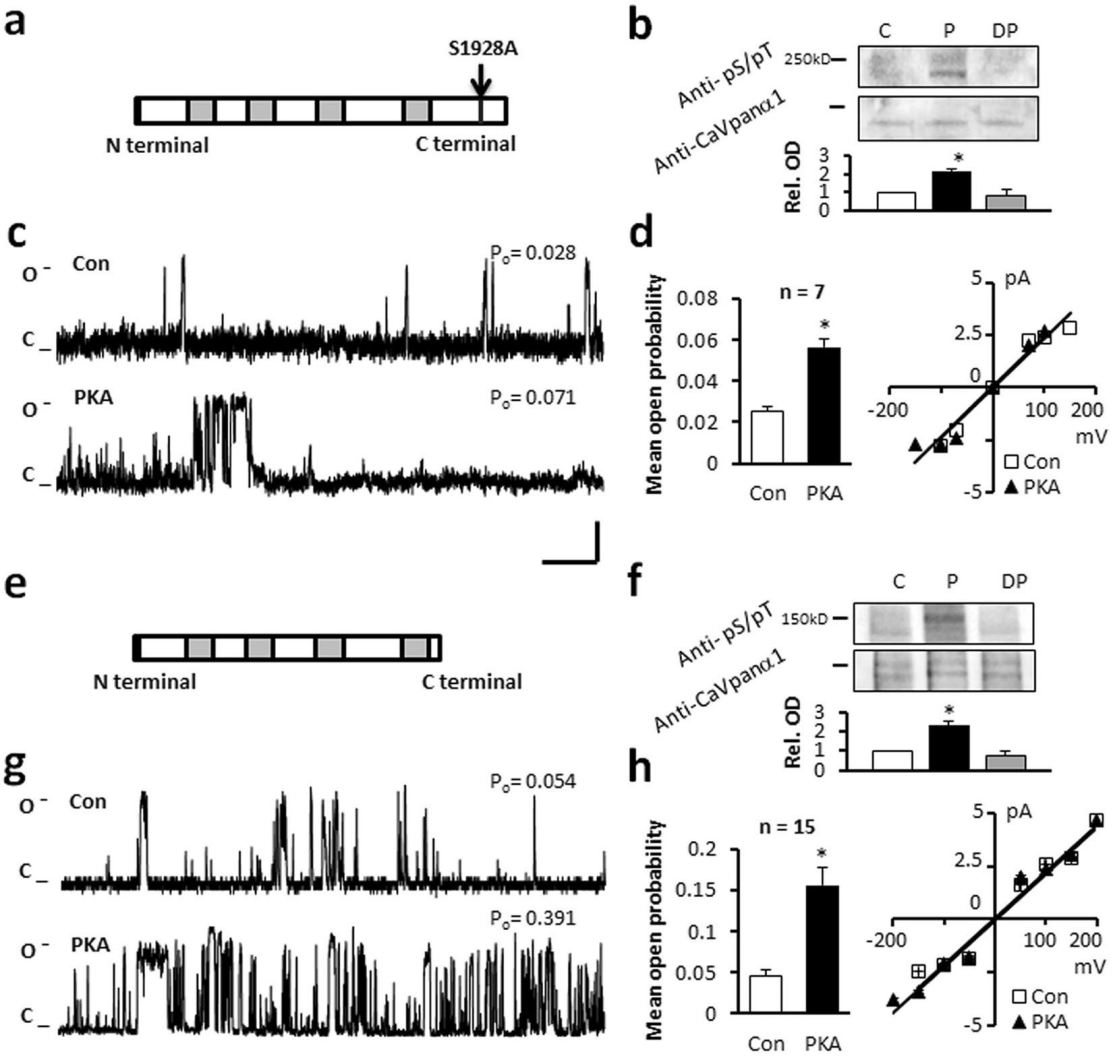
PKA alters the function of the S1928A and truncated C terminal forms of Ca_v_1.2. (**a**) Schematic of S1928A mutant. (**b**) Immunoblot. Relative OD increased after phosphorylation by PKA (P). (**c**) Representative single channel currents recorded at +150 mV in the absence (Con) and presence of PKA. (**d**) I–V relationship (right) and the mean ± SEM channel open probability (P_o_) for currents recorded in control solution or in the presence of PKA. (**e**) Schematic of ΔL1504 truncated C terminal short-NT isoform. (**f**) Immunoblot indicating truncated form at 150 kDa. PKA substrate specific antibody indicating phosphorylation after *in vitro* PKA treatment (P). Full-length blots are presented in Supplementary Figure 2. (**g**) Representative traces (**h**) I–V relationship (right) and the mean ± SEM channel open probability (left) for single channel currents recorded in the absence and presence of PKA. n: number of single channel patches, vertical scale bar: 2 pA, horizontal bar: 100 ms. *p < 0.05 PKA vs Con.

Since there has been significant controversy over the importance of Ser1928 in earlier studies, we tested the Ser1928Ala mutant version of the long-NT isoform of Ca_v_1.2 channel. Results from single channel experiments and from semi-quantitative western blot analysis showed no significant difference in the responses measured in mutated and non-mutated protein following application of PKA (Fig. 2a–d, Table 1).

Previous *in vitro* and *in vivo* studies have demonstrated an autoregulatory effect of the distal C terminus of the Ca_v_1 channel, reporting that the C terminus imposes an inhibitory effect on channel currents^4,35,36^. Others reported that the truncation of the distal C terminus of Ca_v_1.2 at Glycine 1796 results in embryonic lethality in mice^15^. To determine whether the C terminus contained the critical serines, we engineered the short-NT channel protein without the C terminus (Fig. 2e) by truncating the protein at leucine 1504. Upon voltage-step to 150 mV we measured a P_o_ of 0.045 ± 0.007 (n = 15) for the C terminal truncated channel (Fig. 2g,h). This is significantly larger than the P_o_ recorded in the long-NT isoform (p < 0.05; Fig. 1). Following application of PKA, the P_o_ further increased to 0.156 ± 0.022 (n = 15; Table 1). There was no change in current-voltage relationship (Fig. 2h), amplitude or mean open dwell time (Table 1) after application of PKA. Truncation of the C terminus of Ca_v_1.2 channel results in altered channel function similar to BayK activation, increase in the open probability (P_o_ = 0.037 ± 0.007, n = 5 vs 0.004 ± 0.003, n = 6 long-NT isoform) as well as the incidence of mode 2 openings of the channel (35.2 ± 8.3% vs 4.7 ± 2.4% long-NT isoform) in the absence of BayK. This supports a role for the intracellular C terminus in channel gating, supporting mode 0 and mode 1 openings.

Immunoblot analysis did not detect a difference in phosphorylation between the full length or truncated isoforms of the channel (normalized relative OD = 2.3 ± 0.2, Table 2).

Our data confirm that truncation of the C terminus releases an inhibitory effect of the C terminal domain on channel gating but the C terminus does not contain the functional PKA phosphorylation site.

### Protein kinase A cannot phosphorylate the quadruple mutant (S436A + S754A + S834A + S1458A) of truncated C-terminal Ca_v_1.2 channel

The long-NT isoform of Ca_v_1.2 contains 158 serine and 112 threonine residues in total. Of these 108 serines and 65 threonines are located in intracellular regions. The C truncated protein contains 89 serine and 75 threonine residues in total, with 42 serines and 25 threonines located intracellularly according to the predicted membrane topological organization of the channel. Comparative studies of available phosphorylation databases (Kinexus PhosphoNET, PhosphoSitePlus, PHOSIDA) and trials of in silico phosphorylation site prediction programs (NetPhosK 1.0, NetworKin, pkaPS, GPS 2.1, PPSP, Scansite^37,38^) were used to predict PKA substrate sites. Considering direct enzyme-substrate interactions when a serine was preceded by an arginine at −3 position, serine 436 (homologous to S465 in Q13936-1) in the Repeat I-II linker, serine 754 (S783) and serine 834 (S863) in the Repeat II-III linker region, and serine 1458 (S1535) in the C terminal end before the truncation site were mutated to alanine in order to prevent potential phosphorylation of the serine *in vitro*.

**Figure 3:**
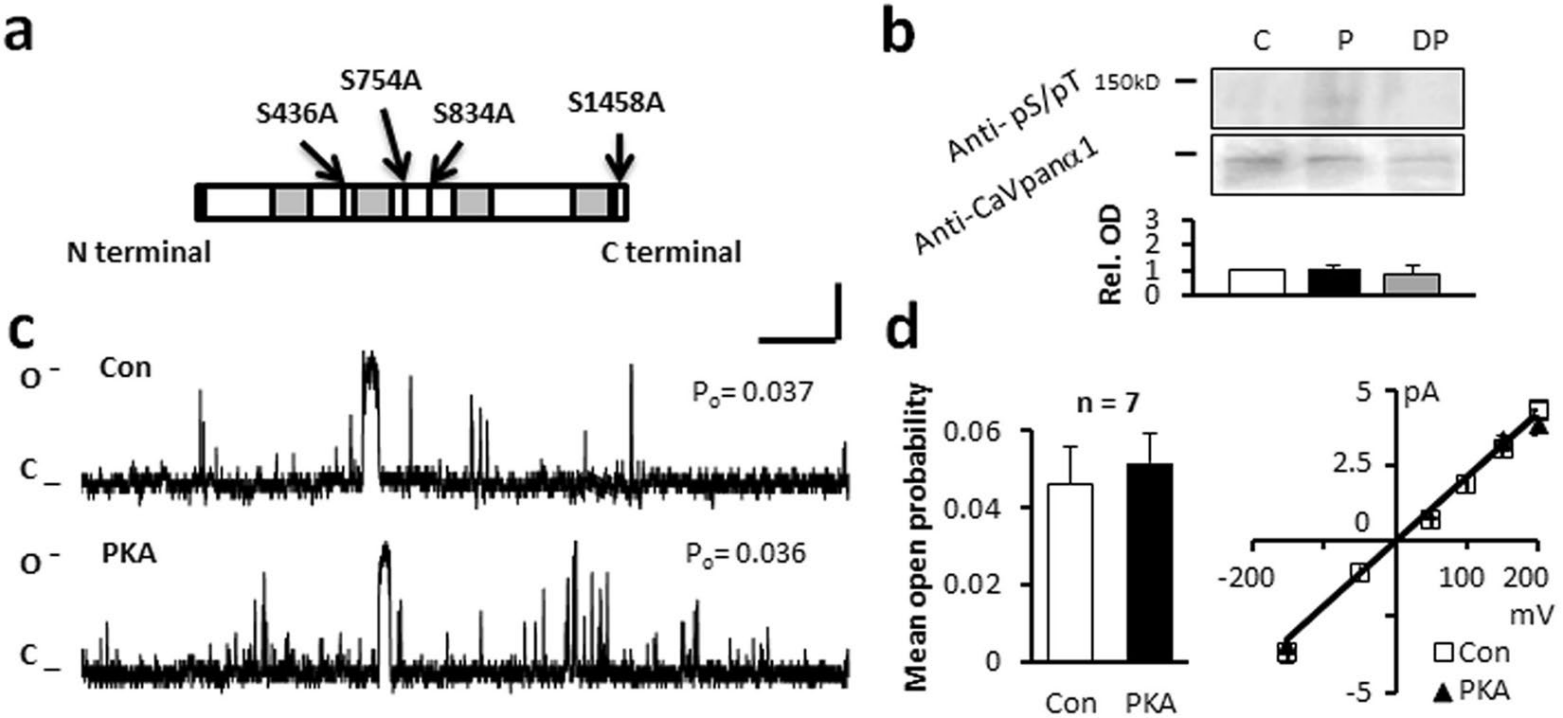
Mutation of four serines in the linker I-II, linker II-III and C terminal end of the truncated C terminus short-NT isoform alters the response of the channel to PKA. (**a**) Schematic of the truncated C terminus isoform indicating mutation of serines at position 436, 754, 834, 1458. (**b**) Western blot analysis could not detect a change in relative optical density after *in vitro* PKA phosphorylation (P), or PP2A treatment (DP) compared with control (C). Full-length blots are presented in Supplementary Figure 2. (**c**) Representative single channel currents of the quadruple mutant recorded at +150 mV in the absence (Con) and presence of PKA in the same patch as shown. (**d**) I–V relationship (right) and the mean ± SEM channel open probability (P_o_) for currents (left) recorded in the absence and in the presence of PKA. n: number of single channel patches, vertical scale bar: 2 pA, horizontal bar: 100 ms.

First we constructed the quadruple mutant, with point mutations at all the potential PKA phosphorylation sites (Fig. 3a). We found no differences in channel activity (P_o_ = 0.046 ± 0.01, n = 7), current amplitude (2.5 ± 0.2 pA) or dwell time (21.5 ± 3.1 ms, Table 1, Fig. 3c,d) under control conditions, and furthermore, no significant changes in channel characteristics were recorded in the presence of PKA. Normalized OD did not demonstrate differences between protein samples with or without PKA treatment (Fig. 3b and Table 2).

### Mutation of serines in the Repeat I-II and II-III linker regions in the truncated C terminal Ca_v_1.2 channel does not alter the response of the channel to PKA phosphorylation

**Figure 4:**
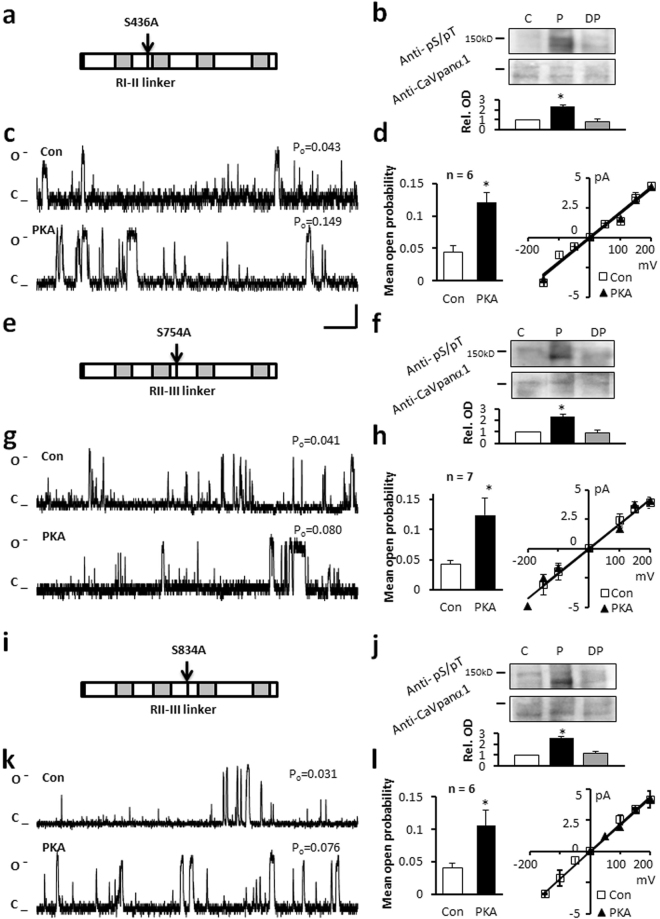
Mutation of serines in the cytoplasmic loop I-II linker or II-III linker region does not alter the response of the channel to PKA. (**a**) Schematic of the S436A mutant isoform. (**b**) Western blot analysis indicates an increase in relative OD after PKA phosphorylation (P). (**c**) Representative single channel currents, (**d**) I–V relationship (right) and the mean ± SEM channel open probability (P_o_) for currents recorded in control solution and in the presence of PKA (left). (**e,i**) Schematic of the truncated C terminus isoform indicating mutations of serine 754 and 834 in RII-III linker region. (**f,j**) Semi-quantitative western blot analysis shows an increase in relative optical density after phosphorylation (P). Full-length blots are presented in Supplementary Figure 2. (**g**,**k**) Representative single channel currents (**h**,**l**) I–V relationship (left) and the mean ± SEM channel open probability (right) for currents recorded in control solution or in the presence of PKA. n: number of single channel patches, vertical scale bar: 2 pA, horizontal bar: 100 ms. Single channel currents recorded at +150 mV. All single channel currents were recorded at +150 mV. *p < 0.05 PKA vs Con.

Mutation of serine 436 to alanine in the Repeat I-II linker region in the truncated C terminal protein (Fig. 4a) resulted in similar P_o_ values to those measured in the wild-type (WT) truncated C terminal Ca_v_1.2 channel, when voltage was stepped to 150 mV (Fig. 4c,d and Table 1). After application of PKA, the P_o_ significantly increased from 0.045 ± 0.009 to 0.121 ± 0.016 (n = 6), without altering the current-voltage relationship, amplitude or mean open dwell time (Table 1). Semi-quantitative densitometry showed a 2.2 ± 0.1 fold increase in phospho-Ser/Thr PKA substrate specific antibody labelling (Fig. 4b, Table 2).

Similar results were obtained when serines at 754 and 834 in the Repeat II-III linker were mutated to alanines (Fig. 4e–l). When the mutated channel protein was voltage-stepped to 150 mV, P_o_ was similar to the WT truncated C terminal protein. Application of PKA increased P_o_ from 0.043 ± 0.007 to 0.124 ± 0.029 (n = 7) and 0.040 ± 0.007 to 0.105 ± 0.025 (n = 6) respectively, without altering other characteristics (Fig. 4g,h,k,l, Table 1). After *in vitro* PKA treatment the relative optical density for PKA substrate specific antibody labelling was increased from 1.0 ± 0.1 to 2.5 ± 0.1 (Fig. 4f,j, Table 2), in a similar manner to the WT form.

Cytoplasmic linker I-II and linker II-III regions of Ca_v_1.2 channel were produced as GST fusion small peptides and used for immunoblot studies. No phospho-serine/threonine PKA substrate specific antibody labelling could be detected suggesting the specified regions do not contain the residues for PKA modification.

### Serine 1458 is responsible for PKA modification of the channel

**Figure 5:**
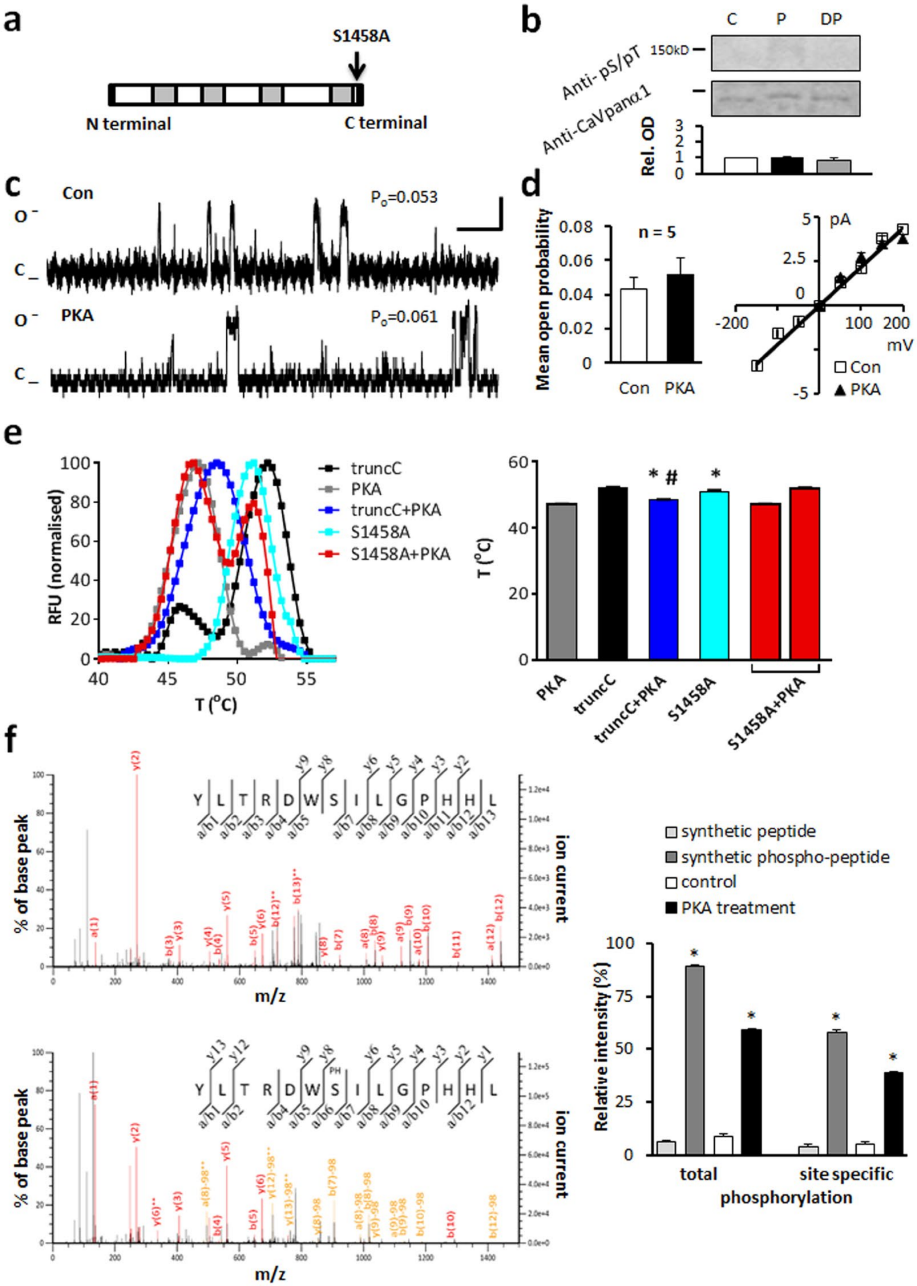
Serine 1458 is responsible for altered function after PKA phosphorylation of Ca_v_1.2 channel. (**a**) Schematic of the truncated C terminus short-NT isoform indicating mutation of serine 1458. (**b**) Western blot analysis could not detect a change in phosphorylation after *in vitro* PKA (P), or PP2A (DP) treatment. Full-length blots are presented in Supplementary Figure 2. (**c**) Representative single channel currents recorded at +150 mV in the absence and presence of 0.5 µM PKA in the same patch. (**d**) I–V relationship (right) and the mean ± SEM channel open probability (P_o_) for currents (left) recorded in control solution and in the presence of PKA. (**e**) Thermal stability assays performed on PKA catalytic subunit, the non-mutated and the S1458A mutated truncated C isoform in the presence or absence of PKA. Note that two peaks were observed after PKA treatment of S1458A mutant (last two bars), corresponding to the control protein and PKA. Mean ± SEM of temperatures for unfolding of proteins as indicated at right. *p < 0.05 truncC + PKA and S1458A mutated truncC vs truncC, ^#^p < 0.05 truncC + PKA vs PKA; (**f**) Representative MS spectra of synthetic YLTRDWSILGPHHL peptide, corresponding to aa1452–1465 of the short N terminal form of Ca_v_1.2 channel (at top) and the same peptide after PKA treatment. Right panel shows semi-quantitative analysis of MS data.

We mutated serine 1458 to alanine located in the proximal carboxyl terminus just before the truncation site in the C truncated form of the channel (Fig. 5a). Under control conditions, the channel function was not altered (Table 1 and Fig. 5c,d). After voltage-stepping to 150 mV we recorded a P_o_ of 0.043 ± 0.007 (n = 5). Application of PKA had no significant effect on the P_o_ or current-voltage relationship (Po = 0.052 ± 0.01, Fig. 5d). Mean open dwell time and single channel current amplitude were also unchanged following PKA treatment.

Western blot analysis did not demonstrate phospho-specific alteration neither in reducing and denaturing (Fig. 5b, Table 2) nor in native conditions (Suppl Fig. 1c). PKA treatment did not changed the mobility of S1458A mutant truncated C terminal form of human Ca_v_1.2 protein as it did in the case of the non-mutated protein (Suppl Fig. 1b,c).

Thermal stability shift analysis is a useful tool for examining protein folding properties, or binding interactions in proteins^39,40^. We performed *in vitro* thermal stability assays on the truncated C terminal short-NT isoform and the S1458A mutant isoform. The melting temperature (Tm) of WT and mutant proteins was determined by taking the temperature at which the RFU (relative fluorescence unit) reached 50% of the maximum. The WT truncated C terminal short-NT isoform and the S1458A mutant isoform demonstrated similar melting peak profiles (Fig. 5e). Following 1 hr *in vitro* PKA treatment the melting temperature of the WT isoform was altered, but not the mutant isoform, demonstrating that protein-protein interactions between PKA and the channel protein had occurred.

Mass spectrometry analysis was performed to demonstrate that serine 1458 could be phosphorylated by PKA. A synthetic peptide of the sequence YLTRDWSILGPHHL, corresponding to aa 1452–1465 of the short N terminal form of Ca_v_1.2 channel was produced^29^. We performed *in vitro* PKA phosphorylation of the non-phosphorylated synthetic peptide for 1 hr at 37 °C and then performed MS/MS analysis of the control and phosphorylated peptides as representative spectra showed on Fig. 5f. To calibrate our experimental system, we also performed MS/MS analysis of a phosphoserine containing version of the synthetic peptide.

Peptide ions for YLTRDWSILGPHHL were observed with a mass of 1706.86 (854.44 2+ m/z) in control samples and 1786.84 (596.62 3+ m/z) in PKA treated samples, showing a 79.98 mass difference, consistent with peptide phosphorylation. A neutral loss of 97.97 from the serine 7 residue was observed in MS/MS spectra of the PKA treated peptide and this was confirmed by *b* and *y* series ions. It is consistent with the loss of HPO_3_ and dehydration of the serine. In the control samples no neutral loss was associated with the residue and *b* and *y* ion series were fully resolved (Supplementary Table 1a and b).

For more detailed analysis we performed semi-quantitative MS analysis on data collected from triplicate MS runs. The data was filtered for the YLTRDWSILGPHHL peptides and grouped by their treatment after Mascot search against the Human-Uniprot database. The relative phosphorylation was calculated by dividing the number of peptide counts showing either total phosphorylation or site specific serine phosphorylation by the total number of peptide counts. Our data showed close correlation between *in vitro* phosphorylated YLTRDWSILGPHHL peptide and the synthetic phospho-peptide (59.1% ± 0.5 vs. 89.6% ± 0.5 relative intensity of total phosphorylation and 39.0% ± 0.5 vs. 58.2% ± 0.8 relative intensity of serine phosphorylation, respectively, Fig. 5f, right panel; Table 2).

### Direct phosphorylation of Ca_v_1.2 channel does not require AKAP

**Figure 6:**
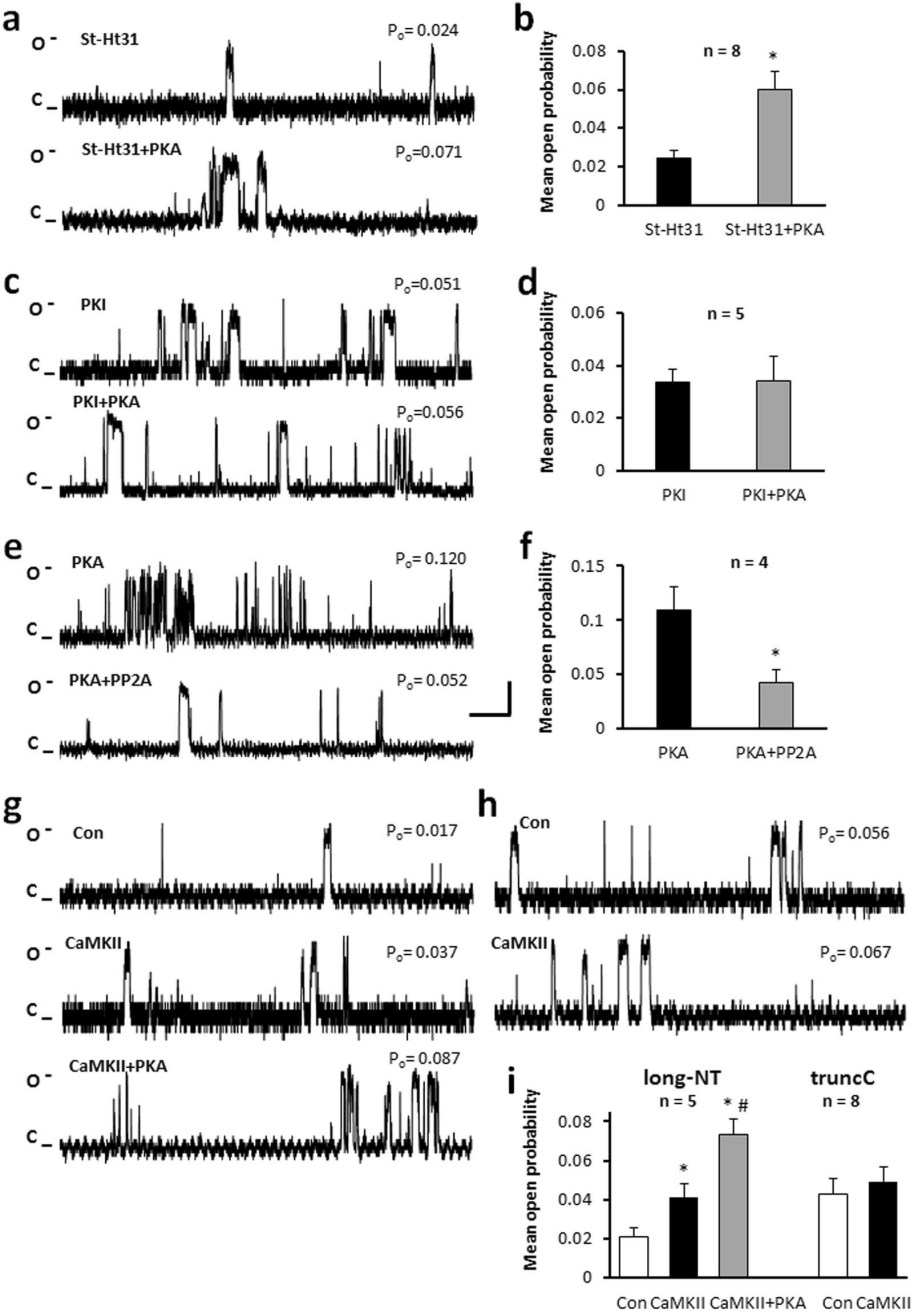
An AKAP is not necessary for direct phosphorylation of Ca_v_1.2 while PKI and PP2A attenuate the effect of PKA. (**a**) AKAP inhibitor St-Ht31 (10 μM) did not alter the effect of PKA on single channel currents of long-NT isoform and the mean open probability (**b**) for currents recorded in the absence and presence of PKA. *p < 0.05 St-Ht31 + PKA vs St-Ht31. (**c**) Representative single-channel currents and P_o_ calculated as mean ± SEM (*d*) in the absence and presence of 5 μM PKI and 5 μM PKI + 0.5 µM PKA in the same patch. (**e**) Representative single channel currents of the C truncated isoform and calculated mean ± SEM channel open probability (**f**) recorded in the presence of 0.5 µM PKA followed by addition of 0.5 µM PP2A in the same patch. *p < 0.05 PKA + PP2A vs PKA alone. To assess effects of CaMKII phosphorylation on long-NT isoform (**g**) and truncated C short-NT isoform (**h**) we substituted the recording solution with Cam/CaMKIIδ (CaMKII). (**i**) Mean ± SEM channel P_o_. Con: control, treatments display as stated in text, n: number of single channel patches, vertical scale bar: 2 pA, horizontal bar: 100 ms. Single channel currents recorded at +150 mV. *p < 0.05 CaMKII vs Con, ^#^p < 0.05 CaMKII + PKA vs CaMKII alone.

In our experimental setting we used heterologously expressed, purified protein and active bovine PKA catalytic subdomains. To rule out the possible effect of an AKAP (that might originate from the cell expression system) on PKA phosphorylation of the channel, we used 10 μM St-Ht31 inhibitor peptide to specifically block the association of the regulatory subunit of PKA and AKAP proteins. Following addition of St-Ht31, PKA significantly increased the function of the long-NT isoform (Fig. 6a,b). We also tested the effect of 1 mM FMP-API-1, a non-specific PKA-AKAP inhibitor^41^. PKA caused a 2.98 ± 0.2 fold increase in P_o_ (Table 1) in the presence of the inhibitor (n = 5) indicating that an AKAP was not required for PKA phosphorylation.

The effect of 5 μM PKI (PKA Inhibitor fragment 5–24 amide) was also examined (Fig. 6c,d). Incubation with the specific antagonist for 30 min completely attenuated the effect of PKA on the function of the truncated C terminal Ca_v_1.2 channel protein (Table 1).

PP2A competes with PKA for the same substrate binding site, so we tested the effect of PP2A preceding or following the PKA application on channel function. Exposure of the truncated C terminal Ca_v_1.2 channel protein to PKA resulted in a significant increase in P_o_ to 0.109 ± 0.021, while subsequent exposure to PP2A decreased P_o_ to 0.041 ± 0.013 (n = 4, Fig. 6e,f, Table 1). No changes in electrophysiological properties were observed. Pre-treatment of the channel with PP2A decreased the P_o_ from 0.043 ± 0.007 to 0.037 ± 0.006 (n = 8; Table 1). Following application of PKA the P_o_ was not significantly altered (0.036 ± 0.009 vs 0.044 ± 0.009; n = 6, Table 1), demonstrating that pre-treatment with PP2A successfully attenuates the effect of PKA.

### The effect of CaMKII on channel phosphorylation

There is significant evidence to suggest that CaMKII can activate the cardiac L-type Ca^2+^ channel by phosphorylating the C terminal end of the pore forming α1 subunit^30^. We examined the effect of CaMKII on the reconstituted long N terminal isoform (Fig. 6g) and the truncated C terminal short N terminal isoform of the Ca_v_1.2 channel (Fig. 6h). Following exposure, CaMKII increased the frequency of single channel openings of the long-NT isoform, but not the truncated C terminal protein (Fig. 6g,h, Table 1), or the S1458A mutated truncated C terminal protein (Table 1). In the presence of PKA, the activity of the long-NT isoform was further increased (Fig. 6g, Table 1), suggesting that the two kinases do not share the same specific phosphorylation sites.

### Semi-quantitative fluorescent phosphoprotein detection

To determine the number of phosphate groups present on the various channel mutants, semi-quantitative fluorescent phosphoprotein detection was performed. All the heterologously expressed channel constructs presented one phosphate group, as expected for posttranslational phospho-modification of heterologously expressed proteins (Table 2). *In vitro* PKA phosphorylation increased the number of phosphate groups to 2, subsequent application of PP2A decreased the value to control levels for each protein, except the S1458A and the quadruple mutant. *In vitro* PP2A treatment alone could not completely dephosphorylate the proteins suggesting that background phosphorylation could have been the result of non-protein kinase A specific posttranslational modifications.

## Discussion

It has been well recognized for decades that activation of the β adrenergic signal transduction pathway is necessary for the protein kinase A-mediated increase in intracellular calcium concentration and increased contraction of the heart. Many groups have implicated direct phosphorylation of the carboxyl terminus of the L-type Ca^2+^-channel α1 C subunit. However, none of the proposed phosphorylation sites on the calcium channel has proven sufficient to increase channel function. The complexities of the experimental approaches and the different models used have made it difficult to resolve the controversies. In our study we used a more direct approach. We expressed and purified the human channel protein and reconstituted the protein into artificial lipid vesicles that allowed us to examine the absolute effect of protein kinase A on the pore forming α1 subunit of the human cardiac L type Ca^2+^ channel using single channel patch-clamp technique.

Computational methods were used to predict possible PKA substrate residues in the intracellular regions of the channel protein and then nominated serine residues were point mutated. Only S1458A and the quadruple mutant (S436A + S754A + S834A + S1458A) of the truncated C terminal short-NT protein were resistant to phospho-modification by cAMP dependent protein kinase. Quantitative fluorescent phosphoprotein analysis detected only one extra phosphate group after *in vitro* phosphorylation by protein kinase A on the long-NT, short-NT and truncated C terminal short-NT channel and the S436A, A754A, S834A mutant forms of the truncated C terminal short-NT protein. There was no significant difference in the number of phosphate groups on the S1458A and the quadruple mutant. These findings indicate that only the serine at position 1458 in the truncated C terminal short-NT isoform (serine 1565 in the long-NT isoform) is the target for PKA phosphorylation. The orthologous serine at 1517 in rabbit Ca_v_1.2 is proposed to be a target for CaMKII phosphorylation^30^. We did not find any evidence for a further increase in channel function by CaMKII in the C truncated Ca_v_1.2 protein (Fig. 6). However we recorded increased open probability in the long NT isoform further validating the C terminus as the functionally significant site for CaMKII phosphorylation.

Although the structure of Ca_v_1.1 was recently determined^42^, the structure of the Ca_v_1.2 protein has not yet been fully resolved. Serine 1458 is located in the proximal carboxyl terminus in close proximity to the Repeat IV S6 membrane spanning region, which is part of the ion conducting pore of the α1 C subunit. Phosphorylation at this site on the proximal C terminus would be expected to induce a local conformational change and add a negative charge close to the ion conducting region, which remains in contact with the rest of the channel following proteolytic cleavage of the distal C terminal domain^4^. The proximal C terminus is also suggested to interact with the N terminus in the folded channel protein.

In the heart adrenergic stimulation is mediated via activation of β1 and β2-AR’s. Both adrenergic receptors are capable of stimulating the adenylate cyclase-cAMP-PKA pathway enhancing Ca_v_1.2 activity, but β1-AR stimulation activates PKA more globally and results in phosphorylation of non-sarcolemmal proteins (such as phospholamban and troponin I), while β2-AR stimulation is more local, limited to activation of PKA compartmentalized with Ca_v_1.2^3,5,43,44^. G protein coupled receptor activation can be further complicated as a result of activation of β3-AR’s that can antagonise β1 and β2-AR signalling via activation of nitric oxide^45^. Compartmentalization is facilitated by proteins such as AKAP’s that assist by directing PKA to target proteins^6,8,9,41^. AKAP’s are proposed to mediate specialised functional responses via specific isoforms. The targeted responses can be directed to compartments within the cell or from cell to cell as is the case with sympathetic innervation of the heart by neurons delivering catecholamines^46^. Recently it was identified that AKAP-mediated PKA responses can be constrained within close proximity of the target^47^. In failing heart, the chronically elevated adrenergic agonist down-regulates the dominant β1-AR and uncouples the β2-AR from downstream Ca^2+^ regulatory target proteins via AKAP signalling complexes^48^. A higher phosphorylation level of E-C coupling proteins is also well documented in heart failure. This results in less efficient excitation-contraction coupling and decreased contractile function^49^, leading to impaired adaptation to hemodynamic demands.

We examined whether phosphorylation of the Ca_v_1.2 channel by PKA alters function and we confirm that direct activation is a requirement for increased open probability. The response does not require the presence of the carboxyl terminal domain and therefore suggests that *in vivo* the physiologically cleaved protein may participate in adrenergic responses. We demonstrate that auxiliary proteins or additional regulatory proteins such as AKAPs are not required for direct activation of the Ca_v_1.2 channel. Our data suggest that *in vivo* auxiliary or regulatory proteins modulate channel function in addition to direct regulation. We have identified the critical serine for the response. We propose that direct phosphorylation of the Ca_v_1.2 by cAMP-dependent protein kinase is sufficient to alter the conformation of the channel and increase calcium influx during sympathetic stimulation.

## Methods

### Heterologous expression and purification of human Ca_v_1.2 protein

In this study two natural variants of the human cardiac L-type voltage-gated Ca^2+^ channel α1 subunit were used, the long N terminal (CAC1C_HUMAN isoform 34, Q13936–34, long-NT), and the short N terminal (short-NT) isoform, which also lacked the exon 45 (CAC1C_HUMAN isoform 18, Q13936–18). Using the short-NT isoform cDNA as a template, S1928A mutant and leucine 1504 deletion mutant (Ctrunc) constructs were generated. The cDNA of the different Ca_v_1.2 α1C forms was cloned into the pcDNA3.1 vector (Invitrogen), and modified to include a HIS_6_ tag at the N terminus.

HEK293T cells (ATCC) were cultured in DMEM medium, supplemented with 5% fetal bovine serum and antibiotics (Invitrogen) at 37 °C and 5% CO_2_. Cell transfection with pcDNA3.1-h CAC1C was carried out using Lipofectamine2000 (Invitrogen) according to the manufacturer’s instructions. In brief, 80–90% confluent cells were incubated for 72 h in the presence of the lipofectamine–plasmid DNA complexes. Serum was added only after the first 6 h incubation. The cell pellet was lysed in lysis buffer (50 mM NaH_2_PO_4_, 300 mM NaCl, 10 mM Imidazole and 1% Tween-20, pH 8.0), centrifuged at 4,200xg for 10 min at 4 °C then purified using Ni-NTA magnetic agarose beads (Qiagen). The purified protein was eluted and stored in −80 °C in elution buffer (50 mM NaH_2_PO_4_, 300 mM NaCl, 250 mM Imidazole and 0.05% Tween-20, pH 8.0) then analyzed by SDS-PAGE and immunoblotting (Suppl. Figure 1a).

### In silico prediction of protein kinase specific phosphorylation sites of Ca_v_1.2 protein

To predict the most probable site for protein kinase A phosphorylation, a comparative study of available phosphorylation databases (Kinexus PhosphoNET, PhosphoSitePlus, PHOSIDA) and trials of in silico phosphorylation site prediction programs (NetPhosK 1.0, NetworKin, pkaPS, GPS 2.1, PPSP, Scansite^37,38^) were used to predict the most likely PKA substrate sites. Considering the probability of direct enzyme-substrate interaction, the intracellularly located serine 436 in the Repeat I-II, serine 754 and serine 834 in the Repeat II-III linker region and serine 1458 in the C terminal end before the truncation site were chosen to be mutated to alanine in order to prevent potential phosphorylation of the –OH group *in vitro* when preceded by an arginine at -3 position.

### Preparation of mutant constructs

Site-directed mutagenesis was undertaken to construct point mutations at serine 436, serine 754, serine 834, serine 1458 using cDNA encoding the ΔL1504 deletion mutant of Ca_v_1.2 as the template using a QuikChange site directed mutagenesis kit (Stratagene). Two oligonucleotide primers containing each desired mutation were extended by Pfu Ultra II DNA polymerase (Stratagene) during PCR. A standard PCR amplification protocol was performed. The quadruple mutant (S436A + S754A + S834A + S1458A) was also constructed from the appropriate mutant cassettes in a stepwise manner. The mutant sequence, orientation, and reading frame of all constructs were confirmed by automated DNA sequencing and restriction digests using *Xba*I and *Bam*HI restriction enzymes (Promega). Mutant DNA was then purified from an overnight culture using low copy plasmid purification protocol (Nucleobond Xtra Midi plus).

### Preparation of cytoplasmic linker I-II and linker II-III region peptide

Since the Repeat I-II as well as the II-III linker region contains a further 7 and 9 serine residues aside from the mutated serines, two small GST-fusion peptide constructs corresponding to the linker regions were also generated and tested for protein kinase A modification.

The cDNA of the truncated C terminal construct of the Ca_v_1.2 channel was used as a template to generate the cDNA fragments corresponding to linker I-II and linker II-III region via PCR amplification using appropriate primers and KOD Hot Start DNA polymerase kit (Merck-Millipore). The linker I-II region sequence incorporating glycine 377 to asparagine 495 or the linker II-III region sequence from aspartic acid 725 to threonine 871 was cloned into *Bam*HI and *Eco*RI sites in pGEX-2T vector with a glutathione-S-transferase (GST) tag. Vector and insert were digested and gel purified, then ligated at a 4:1 ratio using T4 DNA ligase (Invitrogen). Ligated product was transformed into XL-2 blue competent cells (Stratagene). Positive clones were confirmed by Colony PCR and Sanger DNA sequencing. The recombinant pGEX-2T vector was grown in *E. coli* on Luria Bertini-Ampicillin-Chloramphenicol agar plates overnight, and then amplified in liquid media. Once the OD 600 nm reached 0.6, 100 mM IPTG was added to the culture and then incubated for 5 h at 37 °C. The cell suspension was lysed by sonication. The GST fusion peptides corresponding to linker I-II and linker II-III regions of the Ca_v_1.2 channel were then purified by affinity chromatography using glutathione agarose (Sigma), the product was eluted (elution buffer: 10 mM reduced glutathione in 100 mM Tris HCl, ph~7.5) then stored at −80 °C for later use. The purified protein was analyzed by SDS-PAGE.

### Proteoliposome patch clamp

Single channel experiments were performed on liposome-reconstituted purified Ca_v_1.2 channel protein (1:1000 protein/lipid ratio) following the dehydration/rehydration method as previously described^28^. Liposomes were added to the bath filled with solution (200 mM NaCl, 20 mM BaCl_2_ and 5 mM HEPES, pH 7.4) for 10 min at room temperature to promote the formation of blisters. External and pipette solution (100 mM BaCl_2_, 50 mM NaCl and 10 mM HEPES, pH 7.4) contained recording solution with 2 μM BayK8644 (Sigma) to enhance channel openings. The back filled microelectrode had an average resistance of 16–17 MΩ. Single channel currents were filtered at 1 kHz, digitized at 100 kHz, and analyzed using pClamp software (Molecular Devices)^28^. The Ca_v_1.2 channel was determined by the magnitude of the current, changes in open probability of the channel (Po) and sensitivity of the current to the L-type Ca^2+^ channel antagonist nisoldipine. Current traces from control conditions and after addition of 0.5 µM PKA (0.2 U/ml, specific actvity~10 nmol/min/mg, active catalytic subunit bovine; Sigma/Promega) reduced with 1 mM dithiothreitol (DTT, Sigma) and 5 mM N-ethyl-maleimide (NEM, Sigma) substituted with 1 mM ATP-disodium salt, Sigma) treatments within the same patch were compared during analysis. To specifically block the association of the regulatory subunit of PKA and AKAP 10 μM InCELLect™ AKAP St-Ht31 inhibitor peptide (Promega) was used. FMP-API-1 (Sigma) non-specific PKA-AKAP inhibitor was also applied at 1 mM concentration. For direct inhibition of PKA 5 μM PKI (PKA Inhibitor fragment 5–24 amide, Sigma) 30 min incubation was used before PKA treatment. PP2A (Promega), a major phosphatase for L-type Ca^2+^ channels^34^ was used at the same concentration as PKA, to specifically remove the phosphate group from PKA phosphorylated substrate. For CaMKII phosphorylation studies we substituted the recording solution with 12.2 pM (0.2 μg/ml, specific activity 5860 nmol/min/mg) recombinant human CaMKII δ (Abcam) in the presence of 47 nM (0.8 μg/ml) active recombinant human calmodulin protein (Abcam) activated with 200 μM CaCl_2_ and 1 mM ATP disodium salt.

### Western blots

Purified Ca_v_1.2 protein samples and GST fusion cytoplasmic linker I-II and linker II-III peptides were phosphorylated *in vitro* using 1 Unit PKA catalytic subunit (Promega) per 0.5 μg protein for 2 hrs at 37 °C in kinase buffer (50 mM Tris-HCl, 9 mM MgCl_2_, 0.5 mM ATP, pH 7.4, freshly added EDTA free cOmplete mini tablet (Roche), Phosphatase inhibitor cocktail IV and V (Merck Millipore)). Dephosphorylation of the protein samples was achieved by using PP2A (0.05 Unit/reaction Promega) for 2 hrs at 37 °C in phosphatase buffer containing 50 mM Tris-HCl, 150 mM NaCl, 1 mM MgCl_2_, 1 mM DTT, pH 8 with freshly added EDTA free cOmplete mini tablet (Roche). Native PAGE and Western blots were performed to examine protein kinase A phosphorylation over time during *in vitro* treatment (0–60 min), using SDS PAGE but excluding SDS from all solution (Suppl. Figure 1b,c). Purified channel protein (200 nM) or 100 nM purified cytoplasmic linker I-II and linker II-III peptide were incubated with 5X sample buffer (0.125 M Tris-HCl, pH 6.8, 4% SDS, 20% glycerol, 0.5 M DTT and 0.01% bromophenol blue) for 30 min at 45 °C. The channel proteins were separated by 8% SDS-PAGE, the cytoplasmic linker I-II and linker II-III region peptide were separated by 12% SDS-PAGE for 1.5 h at 25 mA constant current per gel. The channel protein was electrophoretically transferred from the SDS-PAGE gel to nitrocellulose membrane using a wet transfer apparatus (Mini-Trans blot, Bio-Rad) in transfer buffer (40 mM Tris-HCl pH 7.4, 20 mM Sodium Acetate, 2 mM EDTA, 0.1% SDS) for 5 h at 40 V, 100 W with constant voltage at 4 °C. After transfer, the membrane was incubated in blocking buffer (5% bovine serum albumin, 0.05% Tween-20 in PBS, pH 7.4) for 1 hr at room temperature. To determine the possible PKA phosphorylation site(s) of purified Ca_v_1.2 channel, phospho-Ser/Thr PKA substrate specific primary antibody (Cell Signaling Technology) was used. To confirm the position of the channel protein on the membrane, the membrane was reprobed after stripping (strip solution: 62.5 mM Tris-HCl pH 6.8, 100 mM β-mercaptoethanol, 2% SDS) with anti Ca_v_1.2 channel primary antibody (anti-Ca_v_pan α1c, Alomone labs) and then goat anti-rabbit HRP conjugate secondary antibody (Abcam). The channel protein was finally detected by ChemiDoc MP Imaging System using Luminata Forte, Western HRP substrate (Millipore). Densitometry analysis was performed on 3 individual immunoblots, using the ImageJ software.

In separate experiments, phosphoprotein specific fluorescent dye was used to detect phosphorylation. After performing staining with fluorescent ProQ Diamond Phosphoprotein Blot Stain (Molecular Probes) to detect phosphorylated molecules, SyproRuby Protein blot stain was used to detect total protein^50,51^. The blots were visualized using ChemiDoc MP. Pro-Q Diamond phosphoprotein blot stain detects phosphoserine-, phosphothreonine- and phosphotyrosine-containing proteins, independent of the sequence context of the phosphorylated amino acid residue. This sensitive technique (detection limit 8–16 ng of phosphoprotein) and signal correlates with the number of phosphate groups, in order to perform quantitative analysis. Subsequently, densitometry was performed for each band on fluorescent images captured by Chemidoc on triplicates, using ImageJ software. Phosphoprotein fluorescent intensity was normalized to total protein fluorescent intensity and then the normalized values were compared with the following protein standards with a known number of phosphate groups (marked for each protein): bovine serum albumin (0), pepsin (1), ovalbumin (2), β-casein (5), α-casein (8) in a concentration range between 10–200 pmol/l^52^.

### Thermal stability assay

Truncated C terminal short-NT isoform S1458A mutant channel was purified from 8% native polyacrylamide gel. The protein band was identified on nitrocellulose membrane after electroblot, using specific anti Ca_v_1.2 channel primary antibody, then the corresponding band was excised from the gel and was cut into fine pieces. The fine pieces of gel were immersed in 0.5 ml ddH_2_O and incubated overnight in a rotary shaker at room temperature. Following incubation, the suspension was centrifuged at 10,000xg for 20 min at 20 °C. The supernatant was transferred into a new tube. Four μg of the purified protein, treated, or non-treated, was mixed with 1:1000 dilution of 5000X Sypro Orange (Molecular Probes). All experiments were performed in triplicates. The samples were heated from 25 °C to 95 °C with a heating rate of 1 °C/min. Protein thermal unfolding curves were monitored by detection of changes in fluorescence of the Sypro Orange dye. The melting temperature (Tm) of the truncated C terminal short-NT isoform S1458A mutant channel proteins was determined by converting the raw fluorescence data to the first derivative of the fluorescence with respect to the temperature (dF/-dT).

### Peptide synthesis

Synthetic peptides of amino acid sequence YLTRDWSILGPHHL, corresponding to aa 1453–1466 of the short N terminal form of Ca_v_1.2 protein were synthesized by Philip Thompson’s laboratory at Monash Institute of Pharmaceutical Sciences, Faculty of Pharmacy and Pharmaceutical Sciences, Monash University using standard Fmoc Solid Phase Synthesis Protocols on 2-chlorotrityl chloride resin. Phosphoserine was coupled as its benzyl protected derivative Fmoc-Ser[PO(OBzl). The peptide-resin was cleaved with 95% TFA: 2.5% dimethoxybenzene: 2.5 triisopropylsilane; and purified by RP-HPLC to > 95% purity as measure by HPLC-MS.

### Mass Spectrometry

Peptide phosphorylation was performed at 37 °C for 1 hour in 50 mM Tris, 10 mM MgCl_2_, 1 mM ATP, 34 mM potassium phosphate, 100 μM DTT, 250 U (~5.35 μg) PKA catalytic subunit (Promega)/5 μg peptide in 25 μl reaction mixture. Synthetic peptides were dissolved in 20 μl of 5% acetonitrile (v/v), 0.1% formic acid (v/v), and 5 μl of this resuspension was loaded onto a C18 polaris high capacity nano LC chip (Agilent) in 95% Buffer A (0.1% (v/v) formic acid in Optima grade water (Fisher)) and 5% Buffer B (0.1% formic acid in Optima grade acetonitrile (Fisher)) using a 1200 series capillary pump (Agilent Technologies). Following loading, samples were eluted from the C18 column and into an inline 6550 Series QTOF mass spectrometer (Agilent Technologies) and separated according to the details outlined in Nelson *et al*. (2014). Agilent .d files were convert to MGF peak lists and were searched against the Human-Uniprot protein database using Mascot 2.3 running on an in-house server, allowing for variable phosphorylation of the protein, no enzymatic digestion, 2+, 3+ and 4+ charge state matches,100 ppm mass tolerance for the parent ion, and 0.5 Da tolerance on MS/MS peaks.

### MS Data semi-quantitative analysis by peptide counts

The Mascot output of the search against the Human-Uniprot database was transformed and analyzed using R (v3.2.2). The data was filtered for the YLTRDWSILGPHHL peptides and grouped by their treatment. The relative phosphorylation was calculated by dividing the number of peptide counts showing a serine phosphorylation by the total number of peptide counts from triplicates of MS measurement analyzed by Mascot (mean ± SEM, n = 3).

### Data availability

In compliance with journal policy we have made data available and archived on a specialised public repository at The University of Western Australia.

## Electronic supplementary material

Supplementary information
