# Author Correction: Identification of a novel cAMP dependent protein kinase A phosphorylation site on the human cardiac calcium channel

**DOI:** 10.1038/s41598-018-24792-3

**Published:** 2018-05-03

**Authors:** Henrietta Cserne Szappanos, Padmapriya Muralidharan, Evan Ingley, Jakob Petereit, A. Harvey Millar, Livia C. Hool

**Affiliations:** 1 School of Human Sciences, University of Western Australia, Crawley, Western Australia Australia; 2 Harry Perkins Institute of Medical Research and Centre for Medical Research, University of Western Australia, Nedlands, Western Australia Australia; 3 School of Veterinary and Life Sciences, Murdoch University, Murdoch, Western Australia Australia; 4 ARC Centre of Excellence in Plant Energy Biology, University of Western Australia, Crawley, Western Australia Australia; 5 Victor Chang Cardiac Research Institute, Darlinghurst, New South Wales Australia

Correction to: *Scientific Reports* 10.1038/s41598-017-15087-0, published online 09 November 2017

The original version of this Article contained a typographical error in the spelling of the author A. Harvey Millar, which was incorrectly given as Harvey A. Millar. This has now been corrected in the PDF and HTML versions of the Article and in the Supplementary Information.

