# Supplementary material for: Identification of a novel cAMP dependent protein kinase A phosphorylation site on the human cardiac calcium channel
Source: Sci Rep. 2017 Nov 9;7:15118. doi: 10.1038/s41598-017-15087-0 (PMC5680263; doi:10.1038/s41598-017-15087-0)

Supplementary information

Identification of a novel cAMP dependent protein kinase A phosphorylation site on the human cardiac calcium channel

Henrietta Cserne Szappanos, Padmapriya Muralidharan, Evan Ingley, Jakob Petereit, A. Harvey Millar, Livia Hool

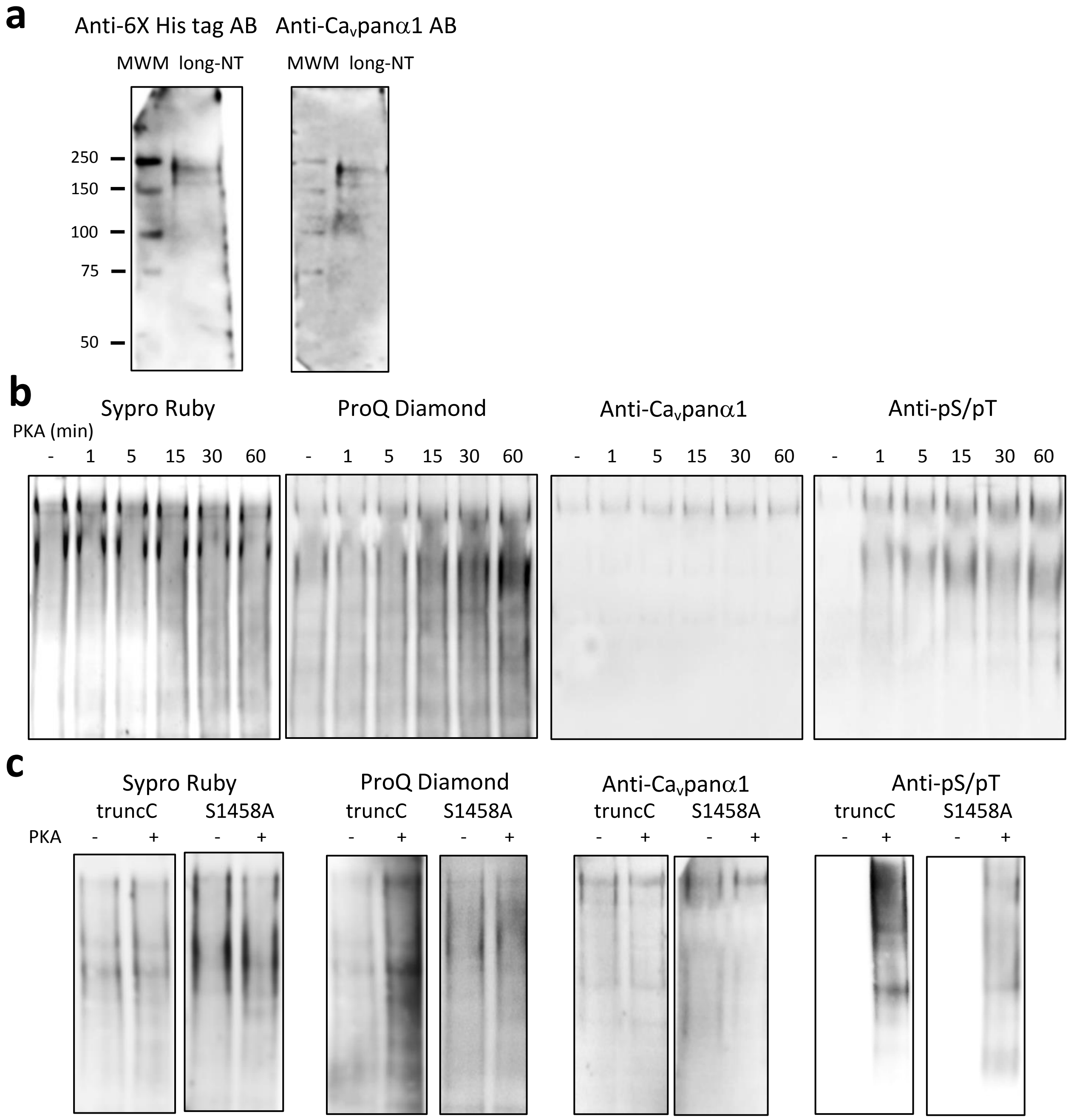

Supplementary Figure 1. (*a*) Full length Western blot images of purified long-NT $Ca_v1.2$ protein probed with anti-6X His-tag specific antibody and channel specific anti-Ca_vpanα1 antibody. (*b*) Native Western blot analysis of truncated C terminal short N terminal $Ca_v1.2$ protein with 0-60 minutes *in vitro* PKA treatment, imaged after total protein and phosphoprotein fluorescent staining, also probed with channel and PKA substrate specific antibodies. (*c*) Differences in phosphorylation levels of S1458A mutated and non-mutated truncated C terminal short N terminal isoforms of the pore forming subunit of cardiac L-type calcium channel with or without 2 hours of *in vitro* PKA treatment revealed on native Western blot analysis.

a

```
Monoisotopic mass of neutral peptide Mr(calc): 1706.8893
Ions Score: 37   Expect: 3.8
Matches : 29/124 fragment ions using 71 most intense peaks   (help)
```

#	a	a^{++}	a^{*}	a^{*++}	b	b^{++}	b^{*}	b^{*++}	Seq.	y	y^{++}	y^{*}	y^{*++}	#
1	136.0757	68.5415			164.0706	82.5389			Y					14
2	249.1598	125.0835			277.1547	139.0810			L	1544.8332	772.9202	1527.8067	764.4070	13
3	350.2074	175.6074			378.2023	189.6048			T	1431.7492	716.3782	1414.7226	707.8649	12
4	506.3085	253.6579	489.2820	245.1446	534.3035	267.6554	517.2769	259.1421	R	1330.7015	665.8544	1313.6749	657.3411	11
5	621.3355	311.1714	604.3089	302.6581	649.3304	325.1688	632.3039	316.6556	D	1174.6004	587.8038			10
6	807.4148	404.2110	790.3883	395.6978	835.4097	418.2085	818.3832	409.6952	W	1059.5734	530.2904			9
7	894.4468	447.7271	877.4203	439.2138	922.4417	461.7245	905.4152	453.2112	S	873.4941	437.2507			8
8	1007.5309	504.2691	990.5043	495.7558	1035.5258	518.2665	1018.4993	509.7533	I	786.4621	393.7347			7
9	1120.6150	560.8111	1103.5884	552.2978	1148.6099	574.8086	1131.5833	566.2953	L	673.3780	337.1926			6
10	1177.6364	589.3218	1160.6099	580.8086	1205.6313	603.3193	1188.6048	594.8060	G	560.2940	280.6506			5
11	1274.6892	637.8482	1257.6626	629.3350	1302.6841	651.8457	1285.6575	643.3324	P	503.2725	252.1399			4
12	1411.7481	706.3777	1394.7215	697.8644	1439.7430	720.3751	1422.7165	711.8619	H	406.2197	203.6135			3
13	1548.8070	774.9071	1531.7805	766.3939	1576.8019	788.9046	1559.7754	780.3913	H	269.1608	135.0840			2
14									L	132.1019	66.5546			1

b

```
Monoisotopic mass of neutral peptide Mr(calc): 1786.8556
Variable modifications:
S7      : Phospho (ST), with neutral losses 97.9769(shown in table), 0.0000
Ions Score: 16   Expect: 3.2
Matches : 32/198 fragment ions using 120 most intense peaks   (help)
```

#	a	a^{++}	a^{*}	a^{*++}	b	b^{++}	b^{*}	b^{*++}	Seq.	y	y^{++}	y^{*}	y^{*++}	#
1	136.0757	68.5415			164.0706	82.5389			Y					14
2	249.1598	125.0835			277.1547	139.0810			L	1526.8227	763.9150	1509.7961	755.4017	13
3	350.2074	175.6074			378.2023	189.6048			T	1413.7386	707.3729	1396.7120	698.8597	12
4	506.3085	253.6579	489.2820	245.1446	534.3035	267.6554	517.2769	259.1421	R	1312.6909	656.8491	1295.6644	648.3358	11
5	621.3355	311.1714	604.3089	302.6581	649.3304	325.1688	632.3039	316.6556	D	1156.5898	578.7985			10
6	807.4148	404.2110	790.3883	395.6978	835.4097	418.2085	818.3832	409.6952	W	1041.5629	521.2851			9
7	876.4363	438.7218	859.4097	430.2085	904.4312	452.7192	887.4046	444.2060	S	855.4835	428.2454			8
8	989.5203	495.2638	972.4938	486.7505	1017.5152	509.2613	1000.4887	500.7480	I	786.4621	393.7347			7
9	1102.6044	551.8058	1085.5778	543.2926	1130.5993	565.8033	1113.5728	557.2900	L	673.3780	337.1926			6
10	1159.6259	580.3166	1142.5993	571.8033	1187.6208	594.3140	1170.5942	585.8007	G	560.2940	280.6506			5
11	1256.6786	628.8429	1239.6521	620.3297	1284.6735	642.8404	1267.6470	634.3271	P	503.2725	252.1399			4
12	1393.7375	697.3724	1376.7110	688.8591	1421.7324	711.3699	1404.7059	702.8566	H	406.2197	203.6135			3
13	1530.7964	765.9019	1513.7699	757.3886	1558.7914	779.8993	1541.7648	771.3860	H	269.1608	135.0840			2
14									L	132.1019	66.5546			1

Supplementary Table 1. MS/MS Fragmentation of YLTRDWSILGPHHL synthetic peptide. Peptide ions for YLTRDWSILGPHHL were observed with mass of 1706.86 (854.44 2+ m/z) in control samples (*a*) and 1786.84 (596.62 3+ m/z) in PKA treated samples (*b*), showing a 79.98 mass difference, consistent with peptide phosphorylation. A neutral loss of 97.97 from the serine 7 residue was observed in MS/MS spectra of the PKA treated peptide and this was confirmed by b and y series ions. It is consistent with loss of HPO_3 and dehydration of the serine. In the control samples no neutral loss was associated with residue and nearly full b and y ion series were resolved.

Supplementary Figure 2. Full length Western blot images corresponding to cropped blot images showed on Fig. 1*b*, Fig. 1*f*, Fig. 2*b*, Fig. 2*f*, Fig. 3*b*, Fig. 4*b*, Fig. 4*f*, Fig. 4*j*, Fig. 5*b*

Full length Western blot images corresponding to cropped blot images showed on Figure 1*b*. Long-NT isoforms of $Ca_v1.2$ protein probed with Anti-pS/pT antibody or channel specific anti-Ca_vpanα1 antibody. C: control, P: in vitro phosphorylated, DP: in vitro dephosphorylated protein. MWM: molecular weight marker, arrow indicates the band corresponding to the channel protein.

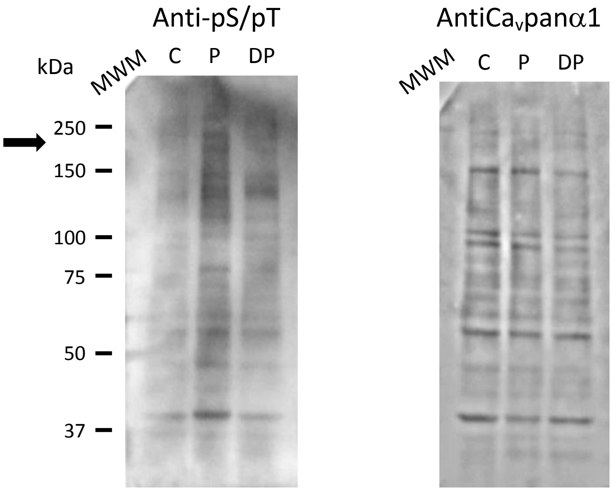

Full length Western blot images corresponding to cropped blot images showed on Figure 1*f*. Short-NT isoforms of $Ca_v1.2$ protein probed with Anti-pS/pT antibody or channel specific anti-Ca_vpanα1 antibody. C: control, P: in vitro phosphorylated, DP: in vitro dephosphorylated protein. MWM: molecular weight marker, arrow indicates the band corresponding to the channel protein. Molecular weight markers usually appeared faintly on immunoblots due to long transfer, corresponding fluorescent image with lower detection limit was used to identify protein bands.

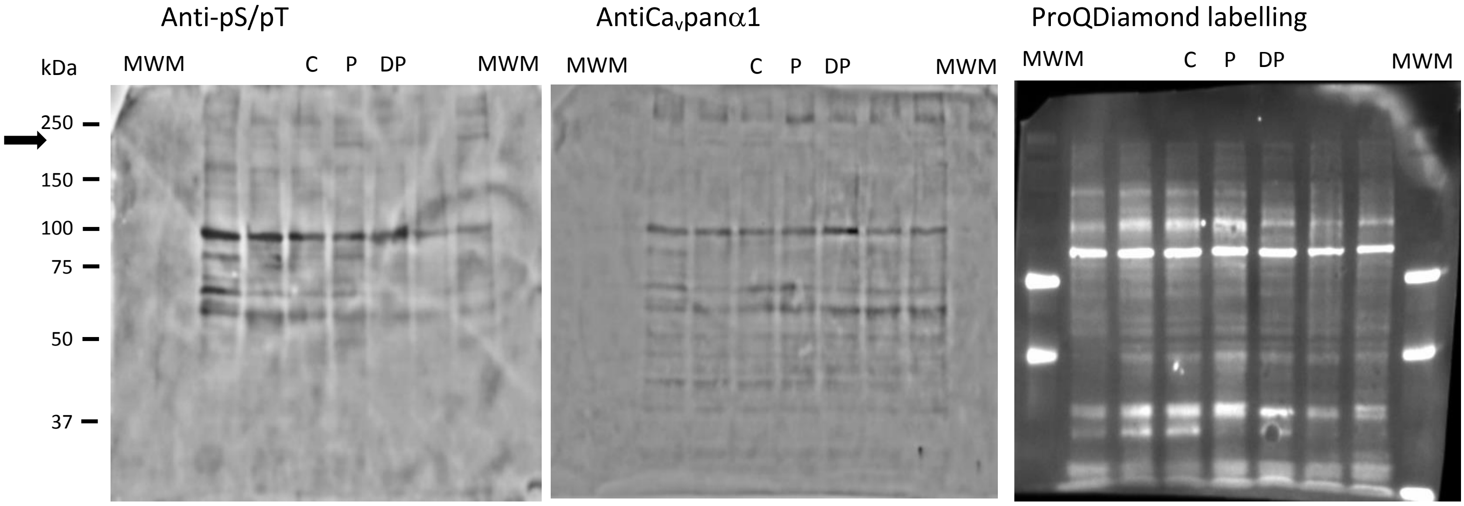

Full length Western blot images corresponding to cropped blot images showed on Figure 2*b*. S1928A mutant version of the long-NT isoform of Ca$_v$1.2 protein probed with Anti-pS/pT antibody or channel specific anti-Ca$_v$panα1 antibody. C: control, P: in vitro phosphorylated, DP: in vitro dephosphorylated protein. MWM: molecular weight marker, arrow indicates the band corresponding to the channel protein.

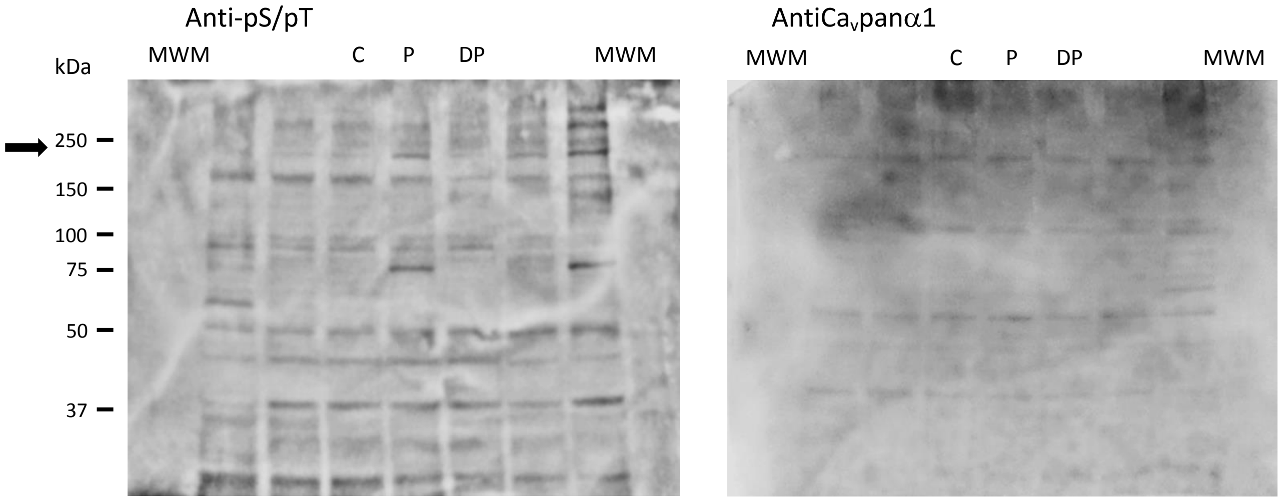

Full length Western blot images corresponding to cropped blot images showed on Figure 2*f*. Truncated C terminus short-NT isoform of Ca$_v$1.2 protein probed with Anti-pS/pT antibody or channel specific anti-Ca$_v$panα1 antibody. C: control, P: in vitro phosphorylated, DP: in vitro dephosphorylated protein. MWM: molecular weight marker, arrow indicates the band corresponding to the channel protein.

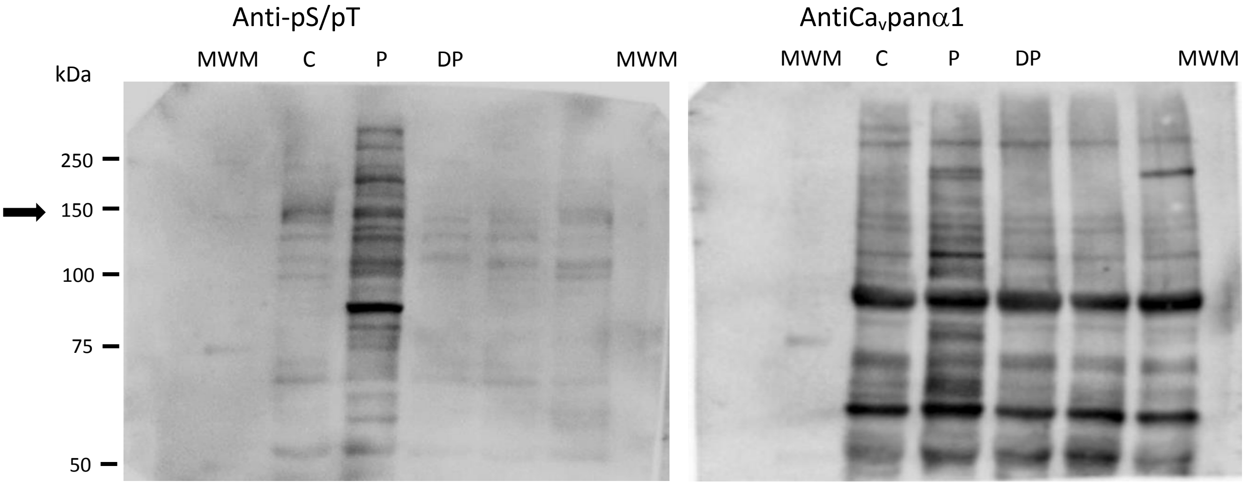

Full length Western blot images corresponding to cropped blot images showed on Figure 3*b*. Quadruple mutant of the truncated C terminus short-NT isoform of Ca_v1.2 protein probed with Anti-pS/pT antibody or channel specific anti-Ca_vpanα1 antibody. C: control, P: in vitro phosphorylated, DP: in vitro dephosphorylated protein. MWM: molecular weight marker, arrow indicates the band corresponding to the channel protein.

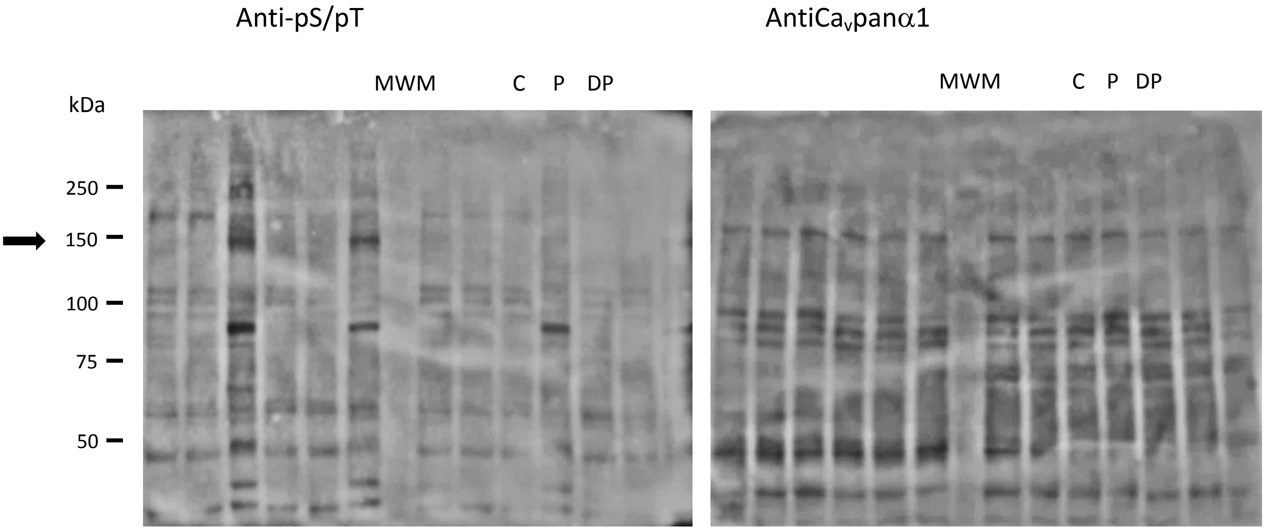

Full length Western blot images corresponding to cropped blot images showed on Figure 4*b*. S436A mutant form of truncated C terminus short-NT isoform of Ca_v1.2 protein probed with Anti-pS/pT antibody or channel specific anti-Ca_vpanα1 antibody. C: control, P: in vitro phosphorylated, DP: in vitro dephosphorylated protein. MWM: molecular weight marker, arrow indicates the band corresponding to the channel protein.

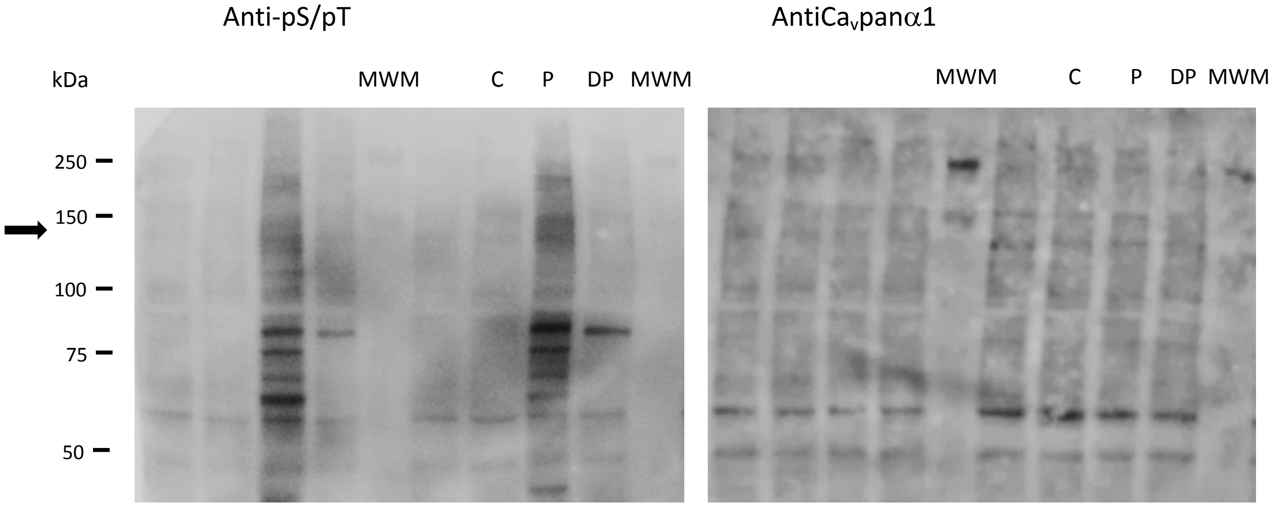

Full length Western blot images corresponding to cropped blot images showed on Figure *4f*. S754A mutant form of truncated C terminus short-NT isoform of $Ca_v1.2$ protein probed with Anti-pS/pT antibody or channel specific anti-Ca_vpanα1 antibody. C: control, P: in vitro phosphorylated, DP: in vitro dephosphorylated protein. MWM: molecular weight marker, arrow indicates the band corresponding to the channel protein.

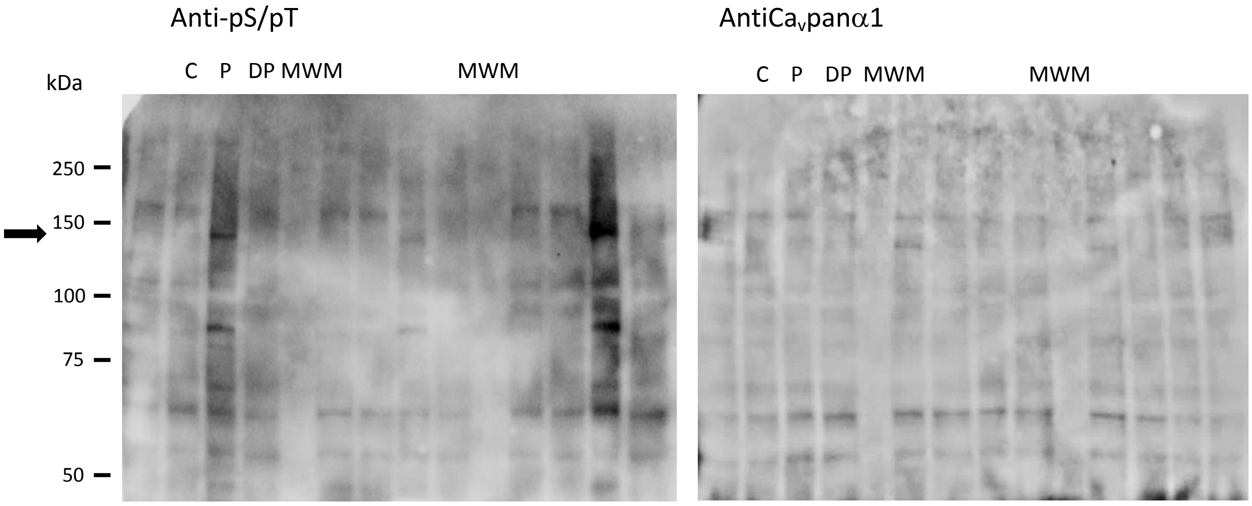

Full length Western blot images corresponding to cropped blot images showed on Figure *4j*. S834A mutant form of truncated C terminus short-NT isoform of $Ca_v1.2$ protein probed with Anti-pS/pT antibody or channel specific anti-Ca_vpanα1 antibody. C: control, P: in vitro phosphorylated, DP: in vitro dephosphorylated protein. MWM: molecular weight marker, arrow indicates the band corresponding to the channel protein.

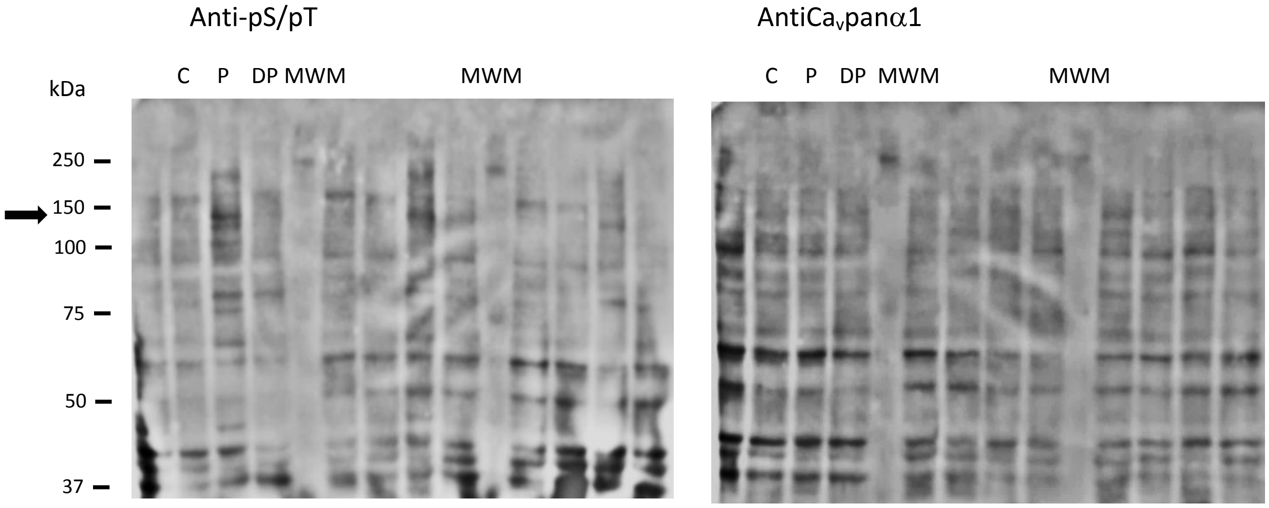

Full length Western blot images corresponding to cropped blot images showed on Figure 5*b*. S1458A mutant form of truncated C terminus short-NT isoform of $Ca_v1.2$ protein probed with Anti-pS/pT antibody or channel specific anti-Ca_vpanα1 antibody. C: control, P: in vitro phosphorylated, DP: in vitro dephosphorylated protein. MWM: molecular weight marker, arrow indicates the band corresponding to the channel protein.

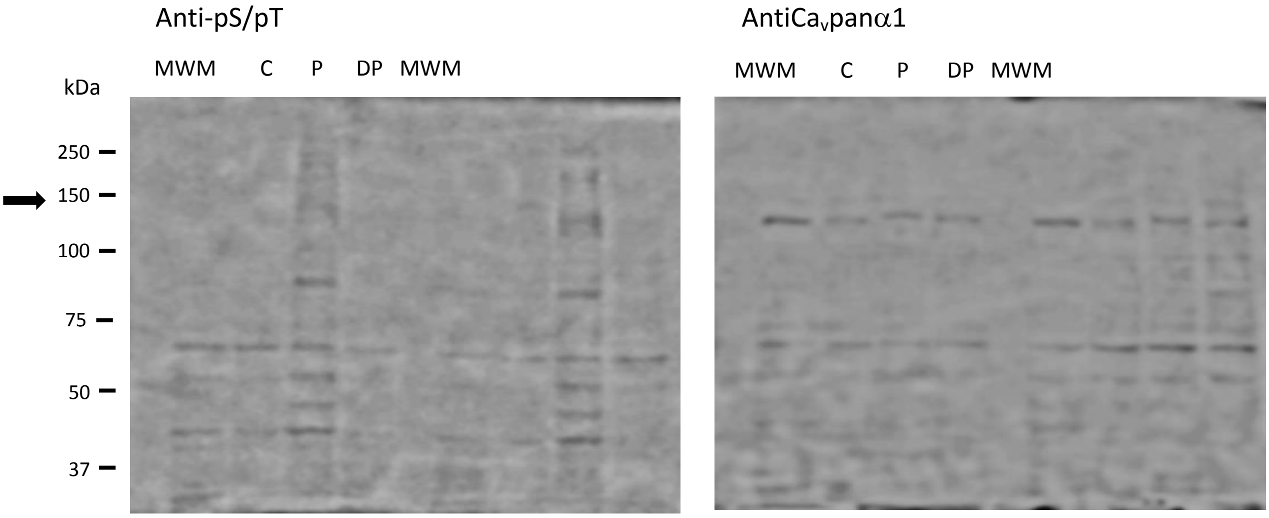

Supplement: Supplementary file 1 — Supplementary information [file 41598_2017_15087_MOESM1_ESM.pdf]
